# Immune cells and inflammatory proteins are differentially associated with subsequent DNA methylation biological aging measures in the Framingham Heart Study Offspring Cohort

**DOI:** 10.1007/s11357-025-01883-4

**Published:** 2025-10-02

**Authors:** Lia Rotti, Joanne M. Murabito, Jiachen Chen, Yumeng Cao, Ahmed A. Y. Ragab, Chunyu Liu, Mengyao Wang, Margaret F. Doyle, Kathryn L. Lunetta

**Affiliations:** 1 Department of Biostatistics, Boston University School of Public Health, Boston, MA USA; 2 Framingham Heart Study, Boston University and National Heart, Lung, and Blood Institute, Framingham, MA USA; 3 Section of General Internal Medicine, Department of Medicine, School of Medicine and Boston Medical Center, Boston University, Chobanian & Avedisian, Boston, MA USA; 4 Department of Pathology and Laboratory Medicine, Larner College of Medicine, University of Vermont, Burlington, VT USA

**Keywords:** Inflammatory biomarkers, Immune cells, Epigenetic aging, Pace of aging

## Abstract

To gain a more comprehensive understanding of the relationships among immunoscenescence and inflammaging, and subsequent epigenetic aging, we measured a panel of 43 immune cell phenotypes and 68 inflammatory proteins collected from blood samples provided by participants in the Framingham Heart Study Offspring Cohort at Exam 7 (1998–2001) and principal component-based DNA methylation (DNAm)-based biologic clocks measured at the subsequent exam (Exam 8 2005–2008), an average of 6 years later. A total of 24 of the 43 immune cell phenotypes and 55 of the 68 inflammatory proteins investigated were significantly associated with at least one of six DNAm aging metrics in age and sex adjusted models. The immune cell associations persisted after accounting for cardiovascular disease and its risk factors, but the protein associations were attenuated. The effects of the inflammatory proteins were larger in the subset of individuals who were < 60 at the protein measurement, compared to those ≥ 60. Immune cell measurements had more associations with age acceleration measures from the 1st generation DNAm clocks and the 2nd generation PhenoAge, while the inflammatory proteins were primarily associated with age acceleration measures based on PhenoAge, GrimAge, and the Dunedin Pace of Aging metric. This study is one of the first attempts to investigate prospective relationships between baseline immune cell composition measured via flow cytometry and inflammatory protein levels and DNAm age measured several years later and represents one of the most comprehensive mappings of immune and inflammatory contributions to DNAm-based age acceleration and pace of aging metrics to date.

**Graphical abstract:**

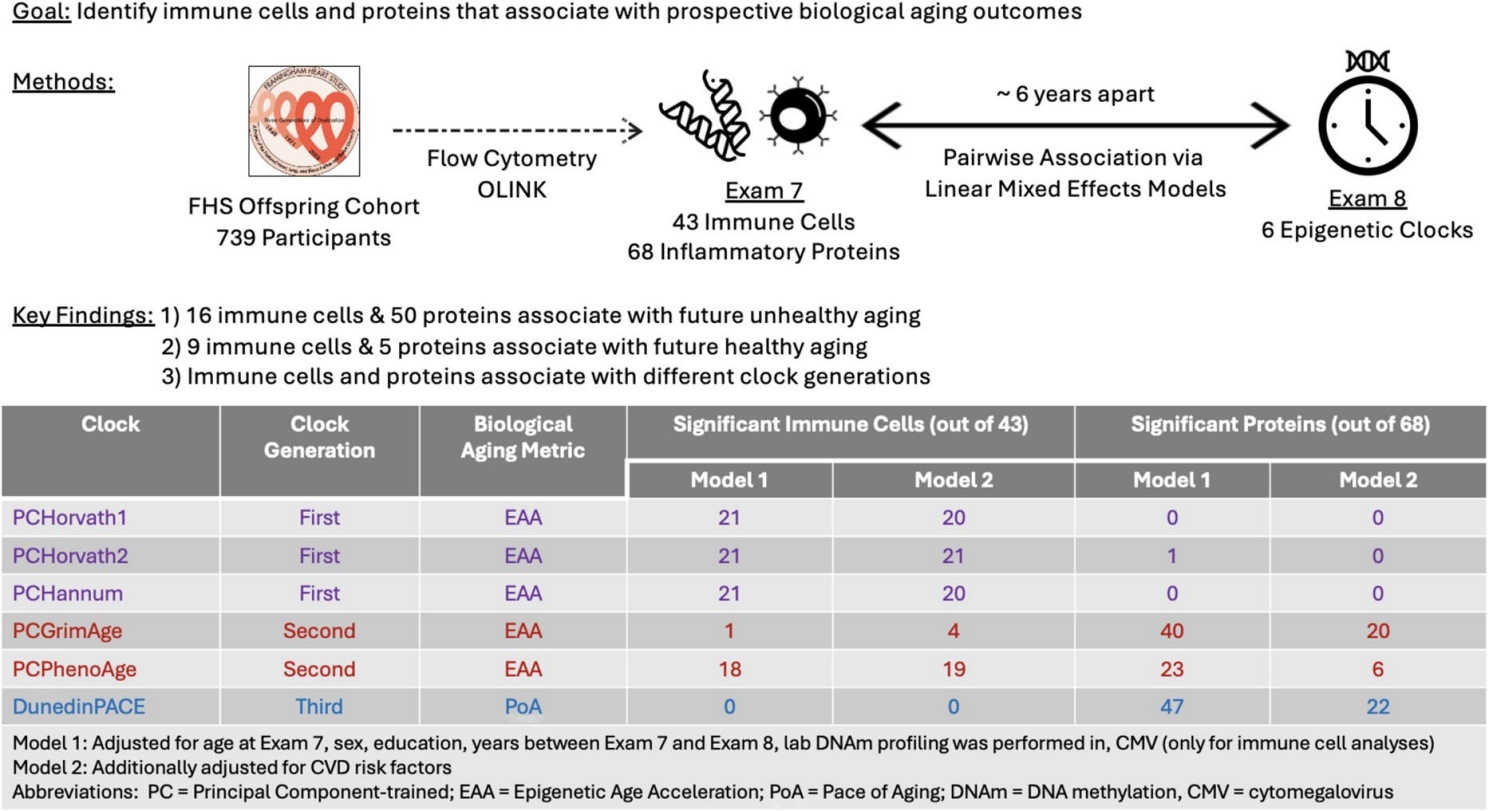

**Supplementary information:**

The online version contains supplementary material available at 10.1007/s11357-025-01883-4.

## Introduction

Chronological age, time since birth, is currently the most well-established risk factor for disease [1]. However, there is notable variability among same-age peers in age-dependent functional decline, disease onset, and associated mortality. The concept of biological aging, which measures cellular and physiological decline over time, can explain these differences between individuals and outperforms chronological age as a determinant of morbidity and mortality [2, 3]. There are several molecular markers of biological aging [4]. Immunosenescence is age-related dysregulation and progressive decline in immune function characterized by a shift in the composition and function of circulating immune cells, including a reduction in naive T cells, an accumulation of memory T cells and B cells, and an expansion of exhausted T cells [5]. Sometimes considered a consequence of immunosenescence, inflammaging is age-related chronic low-grade inflammation characterized by upregulated blood inflammatory protein biomarkers. Perhaps one of the most prominent hallmarks of biological aging in the field of gerontology is predictable age-related changes observed in DNA methylation (DNAm) profiles at cytosine-phosphate-guanine sites (CpGs) across the genome, called epigenetic or DNAm aging. Despite the well-documented identification and characterization of immunologic, inflammatory, and epigenetic cellular changes associated with biological aging, limited research has investigated their interplay [6]. However, recent improvements in methods of measuring immune cell phenotypes and inflammatory markers and the evolving development of epigenetic clocks, algorithms that use an individual’s DNAm profile at select CpGs to estimate their biological age (DNAm age) [7–12] or pace of biological aging through epigenetics [13], provide promising avenues for investigating these relationships and identifying immune and inflammatory biomarkers that associate with and predict healthy biological aging.

The association of “epigenetic age acceleration” (EAA), the difference between epigenetic clock predicted age and chronologic age, and a limited set of immune cell counts imputed from DNAm have been explored using first- and second-generation clocks [10, 11, 14, 15]. These cross-sectional studies have established that EAA from the first-generation Horvath [7] multi-tissue (Horvath1) and Hannum [9] and second-generation PhenoAge [10] and GrimAge [11] clocks have negative correlations with B cells, CD4 + T cells, and CD4 + and CD8 + naïve cells and positive associations with plasmablasts and monocytes. Associations with other immune cell types (granulocytes, NK cells, CD8 + T cells) differed for first- versus second-generation clocks. The principal component (PC)-based versions of these EAA measures have not been tested for association cross-sectionally with immune cell abundance, but correlations of longitudinal change in the PC-based clocks with change in immune cell abundance tended to be larger in size for the PC-based than the original clocks, with consistent direction of correlation to the cross-sectional associations [12].

EAA has not been widely investigated in relation to inflammatory biomarkers. Standard (low sensitivity) C-reactive protein (CRP) has been shown to be positively associated with greater EAA from the first-generation Horvath1 clock, the Horvath1 adjusted for cell type abundance (Intrinsic EAA, or IEAA) [14], and the Hannum extrinsic EAA (EEAA) clock [16–18], as well as the second-generation PhenoAge and GrimAge EAA clocks [6, 10, 11] in cross-sectional analyses. High-sensitivity CRP is also significantly positively associated with the Hannum EEAA clock [16]. Other inflammatory biomarkers that have shown significant positive cross-sectional associations with DNAm clocks include IL6 with Hannum EEAA [16, 18] and PhenoAge and GrimAge [6]; IL18, CXCL10, and TNF with Hannum EEAA [17, 18]; and IFN-gamma with PhenoAge [6]. One study investigated longitudinal associations between 22 inflammation-related plasma markers and later second-generation PhenoAge and GrimAge EAA [6] after an average of 11 years of follow-up as well as cross-sectionally. The cross-sectional associations with the inflammatory biomarkers, including associations with CRP, IFN-gamma, and IL6, were attenuated and non-significant in longitudinal analyses, suggesting lack of meaningful longitudinal associations for these biomarkers after more than a decade of follow-up.

Thus, prior research has demonstrated that some immune cell counts and inflammatory markers are associated cross-sectionally with DNAm-based biologic aging clocks, hinting at a potential relationship between immunosenescence, inflammaging, and epigenetic aging [6, 10, 11, 16, 18]. However, existing studies looked at a limited number of immune cell phenotypes imputed using DNAm and inflammatory protein biomarkers and used just a few DNAm aging metrics from early versions of the epigenetic clocks that have been observed to have low test–retest reliability to assess epigenetic aging. No studies have looked at associations of immune cells measured via flow cytometry with DNAm biologic age, and it is not clear if longitudinal associations between inflammatory biomarkers and DNAm biologic age would be observed in a shorter follow-up interval. Longitudinal associations between immune cells or inflammatory proteins and the more reliable PC-based DNAm clocks have not been reported.

To gain a more comprehensive understanding of the relationships among immunoscenescence and inflammaging, and subsequent epigenetic aging, in this study, we have measured a panel of 43 immune cell phenotypes and 68 inflammatory proteins collected from blood samples provided by participants in the Framingham Heart Study Offspring Cohort at Exam 7 (1998–2001) and DNAm-based biologic clocks measured at the subsequent exam (Exam 8 2005–2008), an average of 6 years later. Our study employs a broader range of immune phenotypes and inflammatory biomarkers than previous studies to understand the complex systems and processes underlying biological aging. We directly measure immune cell abundance using flow cytometry, rather than imputing from DNAm, and we include several immune cell ratios that have previously been used to study the shift in the immune system with age. Further, we use DNAm aging metrics from three different epigenetic clock generations, employing the recently developed and more reliable versions of the clocks [12, 13], to assess the relationships between the immune system, inflammation, and different facets of biological aging. We investigate the potential role of baseline immune and inflammatory profiles as predictors of future healthy biological aging, to identify potential immunologic anti-aging interventions and therapeutics targeting residual inflammation that can slow biological aging. We aim to (1) identify immune and inflammatory markers that predict future healthy biological aging, as well as those associated with adverse DNAm aging outcomes; (2) identify potential moderators of these associations; and (3) compare the relationships of these biomarkers with different measures of DNAm aging derived from newer, improved versions of epigenetic clocks spanning multiple generations.

## Methods

### Study sample

The FHS is a community-based, multi-generational prospective cohort study initially established to identify determinants of cardiovascular disease and cardiovascular risk factors. The FHS Offspring cohort (*n* = 5214) was recruited in 1971 and consists of the children of the FHS Original cohort and spouses of the Offspring [19]. The Offspring cohort has been examined every 4–8 years since enrollment. All participants provided written informed consent at every attended FHS examination. Study protocol and examinations were reviewed and approved by the Institutional Review Board at Boston University Medical Center.

**Fig. 1:**
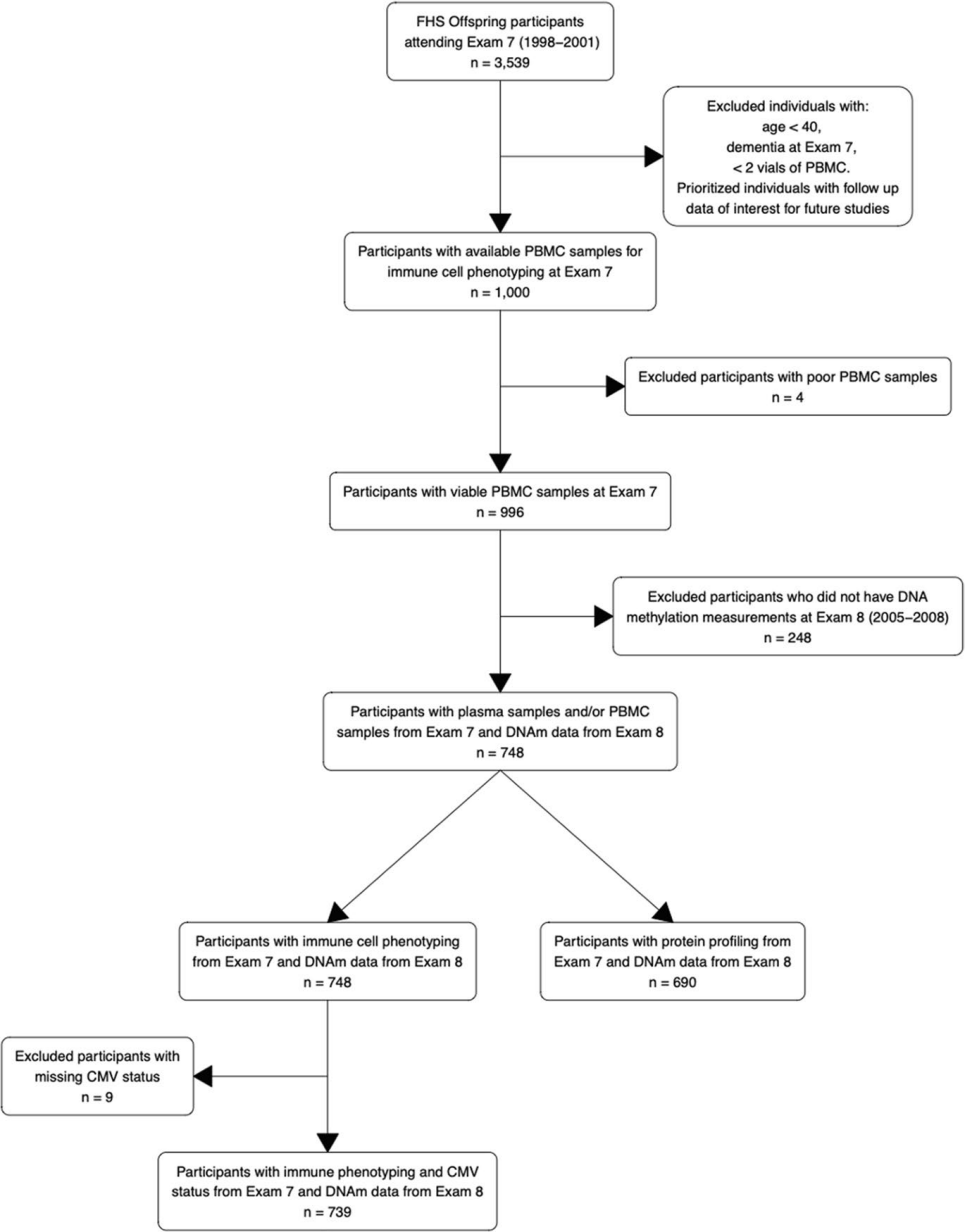
Flow chart for participant inclusion in the immune cell and protein analyses

Sample inclusion and exclusion criteria for the immune cell analyses and protein analyses in this study are shown in Fig. 1. Briefly, we identified 1000 Offspring participants aged 40 years and older who were dementia-free when they attended Exam 7 between 1998 and 2001 and had at least two vials of peripheral blood mononuclear cells (PBMCs) available for immune cell phenotyping. We excluded four samples due to poor PBMC sample quality with missingness in most of the immune cell phenotypes profiled. Among the remaining 996, 748 participants also had DNAm measurements from Exam 8 (2005–2008), which occurred approximately 6 years after Exam 7. A subset of 690 of the 748 individuals additionally underwent protein biomarker profiling with Exam 7 plasma.

For the immune cell analyses, 9 participants with missing cytomegalovirus (CMV) status were excluded, as CMV has a large impact on immune cell abundance and composition (see below). This left a final sample of 739 participants who had immune cell phenotypes and CMV status from Exam 7 and DNAm data at Exam 8. The sample for the protein analyses included a subset of 690 participants from the 739 who also had protein profiles at Exam 7.

### Immunophenotyping

Immunophenotyping methods and quality control procedures for this sample have been described previously [20]. Briefly, PBMCs were isolated from whole blood and were cryopreserved at the FHS Offspring Cohort Exam 7 from 1998 to 2001, which is the first examination with existing stored PBMCs. A total of 116 immune cell phenotypes were profiled from the PBMC samples. All immune cell phenotype data are presented as a percentage of their main parent population. In addition, ratios of immune phenotype measures based on age-related changes of naive, memory, and effector T cell subsets for CD4 + and CD8 + T cells were calculated using the immunophenotyping data [21].

In this study, we selected 43 immune cell phenotypes to use in our analyses. This panel includes 37 immune cell subtypes and six immune cell proportion ratios of T cell subtypes: the ratio of CD4 + cells to CD8 + cells (CD4/CD8 ratio), the ratio of CD4 + T naïve cells to CD4 + T memory cells (CD4 Tn/Tm ratio, defined as CD4 + Tn/(Tcm + Tem + Teff)), the ratio of CD8 + naïve cells to CD8 + T memory cells (CD8 Tn/Tm ratio, defined as CD8 + Tn/(Tcm + Tem + Teff)), the ratio of Granzyme B + CD8 + T cells to Granzyme B + CD4 + T cells (Granzyme B CD8/CD4), the ratio of Th17 cells to regulatory CD4 + T cells (TH17/CD4 Treg ratio), and the ratio of Tc17 cells to regulatory CD8 + T cells (Tc17/CD8 + Treg ratio). The CD4/CD8 ratio is a widely used measure of senescence. The CD4 + Tn/Tm and CD4 + Tn/Tm ratios are also considered to be age-related immune cell phenotypes (ARIPs) [21, 22], and the three additional ratios are known to associate with age-related outcomes. Note that we use “CD4” to indicate CD4 + cells, “CD8” to indicate CD8 + cells, and “immune cell phenotypes” to describe the collection of 43 immune cells and ratios throughout. A list of the 43 immune cell phenotypes and their parent cell populations is provided in Tab s1 (Online Resource 1).

### Cytomegalovirus (CMV)

Cytomegalovirus (CMV) is a common pathogen known to associate with alterations to the immune system [5, 23, 24]. CMV IgG avidity was assayed using existing plasma samples from Offspring Exam 7 as described previously [20]. CMV exposure was categorized into seropositive (CMV > 15 U/ml) and negative or equivocal (CMV ≤ 15 U/ml).

### OLINK inflammation panel

Inflammatory biomarker profiling and quality control procedures for this sample have been described previously [25]. Briefly, the OLINK inflammation panel measured 92 inflammation-related human protein biomarkers using existing plasma samples from Offspring Exam 7. Protein levels were expressed by Normalized Protein eXpression (NPX) units, measuring a relative quantification unit on a log_2_ scale, where one NPX difference means a doubling of protein concentration [26]. Proteins with > 50% of the participant values below the limit of detection (LOD) were excluded from analyses, leaving a total of 68 OLINK inflammatory proteins for our analyses, which are listed in Tab s2 (Online Resource 1).

### DNA methylation measurement

DNA methylation (DNAm) profiling and quality control procedures have been described previously [27]. Briefly, DNAm was measured from whole blood taken from Offspring participants attending Exam 8, on average 6 years after Offspring Exam 7, using the Infinium HumanMethylation 450 K BeadChip array (Illumina, Inc., San Diego, CA, USA). The Offspring DNAm levels were quantified in two separate laboratories, either at Johns Hopkins University (JHU-lab) or at the University of Minnesota (UMN-lab). For each laboratory-specific batch of DNAm data, several quality control procedures were applied. They included the removal of cross-reactive probes that mapped to multiple locations at the CpG level, removal of low-quality probes with a missing rate greater than 20%, probes with average detection *p* > 0.01, and probes with single-nucleotide polymorphisms (SNPs) within 10 bp of single-base extension. Participants were excluded from DNAm profiling if they were identified as an outlier by multidimensional scaling (MDS) techniques, they had a missing rate > 1% across methylation probes, or were a poor match to the 65 SNP genotypes on the Infinium HumanMethylation 450 K Beach chip array.

#### Calculation of DNA methylation age acceleration and pace of aging

Five different PC-trained epigenetic clocks and DunedinPACE encompassing three clock generations serve as the primary outcome variables for the analyses. Table 1 lists the clocks and corresponding age metrics employed by our study. For the first- and second-generation clocks, we use the metric commonly called epigenetic age acceleration (EAA), as the DNAm aging metric. A positive EAA, referred to as “accelerated” DNAm aging, indicates a more advanced DNAm age relative to chronological age, while a negative EAA, referred to as “decelerated” DNAm aging, indicates that an individual is biologically younger than their chronological age. The third-generation pace of aging (PoA) metric is measured in years of biological/physiological aging per calendar year. A PoA greater than one indicates that an individual experiences the equivalent of more than 1 year of physiological decline per calendar year, while a PoA less than one indicates “slow” DNAm aging compared to chronologically same-aged peers.

**Table 1:** Description of the Epigenetic clocks and their DNAm Aging Metrics

Epigenetic Clock	Generation	# of CpG (PCs)	Sample Source	DNAm Aging Metric
PCHorvath1	First	121	Blood, Tissues	EAA.PCHorvath1
PCHorvath2	First	140	Blood, Tissues	EAA.PCHorvath2
PCHannum	First	390	Blood	EAA.PCHannun
PCPhenoAge	Second	652	Blood	EAA.PCPhenoAge
PCGrimAge	Second	1934	Blood	EAA.PCGrimAge
DunedinPACE	Third	173	Blood	PoA.DunedinPACE
Note: PC-prefix indicates that the clock was trained on CpG principal components				

The PCHorvath1, PCHorvath2, PCHannum, PCPhenoAge, and PCGrimAge clocks and their EAA metrics were calculated using code publicly available on GitHub at https://github.com/MorganLevineLab/PC-Clocks [12]. The DunedinPACE clock and its PoA metric were calculated using code publicly available on GitHub at https://github.com/danbelsky/DunedinPACE [13]. EAA was measured as the residual from regressing DNAm age estimate from first- and second-generation PC-based epigenetic clocks onto chronological age, which ensures that the effect of “healthy biological aging” can be examined independently of chronological age. PoA estimates were directly output from the third-generation DunedinPACE clock, requiring no residual calculation like EAA. We use “DNAm aging metrics” to refer to the collection of EAA and PoA throughout. The clocks and the names for the DNAm aging metrics used in this study are listed in Table 1.

### Statistical analyses

For all association analyses, the immune cell phenotypes, protein biomarkers, and DNAm aging metrics underwent rank-based inverse normal transformations to standardize the distribution to a mean of 0 and standard deviation (SD) of 1, reduce skewness, and facilitate comparison of effect sizes across different immune, inflammatory, and DNAm aging domains.

We tested each of the 68 inflammatory protein biomarkers and 43 immune cell phenotypes (predictors) measured at Exam 7 for prospective association with each of the six DNAm aging metrics (outcomes) measured at Exam 8 individually using linear mixed-effects regression. All regression models accounted for familial relationships via the kinship coefficient matrix. We conducted these association analyses using two sets of covariates in the full sample.

The primary model (Model 1) for the association analyses adjusted for chronological age at Exam 7, sex, education level, time in years between Exam 7 (immune phenotyping and protein profiling) and Exam 8 (DNAm measurements), and lab index for DNAm batch effects. CMV exposure status was included as an additional covariate in the immune cell analyses to control for CMV’s influence on the distribution of T cell subsets and its effects on immunosenescence [5, 24]. CMV is not considered a primary causative factor of inflammaging, and so it was not included as a covariate in the protein analyses [28].

We used a secondary model (Model 2) to assess the robustness of the results after accounting for medical history and a number of cardiovascular disease (CVD) risk factors at baseline (Exam 7). Model 2 incorporated all covariates used in Model 1 as well as (1) indicators for prevalent CVD, prevalent atrial fibrillation (AF), prevalent cancer, and prevalent stroke at Exam 7 and (2) baseline CVD risk factors, including body mass index (BMI, kg/m^2^), smoking status, diabetes status, systolic blood pressure (SBP, mmHg), treatment for hypertension, total cholesterol level (TC, mg/dL), high-density lipoprotein cholesterol levels (HDL, mg/dL), and use of lipid-lowering agents at Exam 7 [20, 25].

Education level was recorded at FHS Offspring exams 2 and 8 and during off-exam-cycle neuropsychological testing. The highest education level reported was categorized into four education levels: attended some high school, high school graduate, attended some college, and college graduate [20, 25]. The presence of diabetes was defined by whether any of the following was satisfied: use of antidiabetic medications, fasting blood glucose level of 126 mg/dL or higher, or random blood glucose level of 198 mg/dL or higher [20, 25]. Prevalent CVD at Exam 7 was determined based on current or previous diagnosis of coronary heart disease (myocardial infarction, angina pectoris, coronary insufficiency), transient ischemic attack, intermittent claudication, and congestive heart failure adjudicated by a panel of senior investigators [20, 25, 29].

Two participants included in both the immune cell phenotype sample and protein subsample were missing BMI at Exam 7 due to missing height measurements. Both participants had weight at Exam 7 available. Height at Exam 7 for the two participants was imputed by taking the average of their height at Exams 6 and 8, allowing for the calculation of imputed BMI at Exam 7.

In addition to the analyses performed in the full sample, we performed stratified analyses by sex (male or female) and dichotomized age group (< 60 or ≥ 60) at Exam 7 due to well-documented sex-based immunological differences [30] and recent research that suggests biomolecular shifts contributing to the biological aging process occur around age 60 [31]. For the immune analyses, we also stratified by CMV status (seropositive or negative/equivocal) since viral infection, particularly from CMV, is known to influence the distribution of immune cells in infected individuals [5, 24].

For all association analyses, we report effect sizes and their 95% confidence intervals (CIs) for each immune cell phenotype or inflammatory protein. Since the outcomes and predictors are standardized, effects are reported as SD units change in the DNAm aging outcome per SD unit increment in the immune cell phenotype or inflammatory protein level. The false discovery rate (FDR) [32] was used to control for multiple testing for each DNAm aging metric outcome and stratum separately, and a FDR $<$ 0.05 was set as the threshold to declare significant associations within each set of analyses. An analysis set was defined by having the same predictor set (immune or protein), same covariate set (Model 1 or Model 2), same sample (full, younger, older, males, females, CMV positive or CMV negative/equivocal), and same DNAm aging outcome, so the BH correction was performed within 68 pairwise associations with one outcome for the protein analyses and 43 pairwise associations with one outcome for the immune analyses.

All analyses were performed using R software version 4.4.0. The linear mixed effects models were fitted using the lmekin function from the coxme package [33].

## Results

### Participant characteristics

Participant characteristics for the full *n* = 739 immune cell phenotype sample, including all individuals appearing in at least one pairwise test for association, as well as in the age strata (< 60 years of age at Exam 7, ≥ 60 years of age at Exam 7), sex strata (males, females), and CMV strata (positive, negative or equivocal) are summarized in Table 2. Among the participants included in the full immune analysis sample, 51% were female. The average age of the participants at baseline (Exam 7) was 60 with a range from 40 to 85 years, and the DNAm age metrics measured at Exam 8 were observed an average of 6 years later. About half of the participants were CMV seropositive. Notably, older participants and females were significantly more likely to be CMV positive. Males and older participants were more likely to be on treatment for hypertension and lipids and have prevalent diseases. The protein analysis subsample (*n* = 690) was a true subset of the protein analysis sample and thus shared similar demographic characteristics. Summaries of the 43 immune cell phenotypes and 68 inflammatory proteins in the full sample and for all strata are in Tab s3 and Tab s4 (Online Resource 1). Pearson correlation plots for the immune cell phenotypes and inflammatory proteins are provided in Fig s1 and Fig s2 (Online Resource 2). For more than half of the immune cell phenotypes, average levels differ by age, sex, and/or CMV status. Likewise, average protein levels differed between age strata for 52 of the 68 inflammatory proteins, with older participants having higher levels for most. For 30 of the 68 proteins, average levels differed between males and females.

**Table 2:** Characteristics for immune cell phenotype sample

		Age Strata		Sex Strata		CMV Strata	
Demographic	All	Younger (<60)	Older (≥60)	Male	Female	CMV Negative or Equivocal	CMV Positive
Sample size, n (%)	739	373 (50.5%)	366 (49.5%)	361 (48.8%)	378 (51.2%)	377 (51.0%)	362 (49.0%)
Female, n (%)	378 (51.2%)	201 (53.9%)	177 (48.4%)	0 (0%)	378 (100%)	184 (48.8%)	194 (53.6%)
Age at Ex7, mean (range)	60 (40, 85)	53 (40, 59)	67 (60, 85)	60 (40, 83)	60 (41, 85)	58 (40, 79)	62 (42, 85)
Years between Ex7 and Ex8, mean (sd)	6 (0.719)	6 (0.729)	6 (0.707)	6 (0.729)	6 (0.709)	6 (0.715)	6 (0.723)
UMN lab, n (%)	588 (79.6%)	330 (88.5%)	258 (70.5%)	252 (69.8%)	336 (88.9%)	308 (81.7%)	280 (77.3%)
CMV positive, n (%)	362 (49.0%)	142 (38.1%)	220 (60.1%)	168 (46.5%)	194 (51.3%)	0 (0%)	362 (100%)
BMI at Ex7, mean (sd)	28 (4.76)	28 (5.05)	28 (4.45)	29 (4.12)	27 (5.24)	28 (4.60)	28 (4.92)
Attended college, n (%)	523 (70.8%)	279 (74.8%)	244 (66.7%)	261 (72.3%)	262 (69.3%)	275 (72.9%)	248 (68.5%)
Current smoker, n (%)	96 (13.0%)	65 (17.4%)	31 (8.5%)	36 (10.0%)	60 (15.9%)	52 (13.8%)	44 (12.2%)
Diabetes, n (%)	65 (8.8%)	23 (6.2%)	42 (11.5%)	37 (10.2%)	28 (7.4%)	33 (8.8%)	32 (8.8%)
SBP, mean (sd)	125 (17.6)	121 (16.3)	131 (17.4)	127 (16.3)	124 (18.5)	124 (17.2)	127 (17.8)
Total cholesterol, mean (sd)	199 (36.6)	201 (36.6)	198 (36.5)	193 (35.4)	206 (36.6)	200 (36.9)	199 (36.3)
HDL cholesterol, mean (sd)	53 (16.6)	54 (16.2)	53 (16.9)	46 (12.3)	61 (16.6)	53 (16.6)	53 (16.5)
Hypertension Rx, n (%)	224 (30.3%)	75 (20.1%)	149 (40.7%)	132 (36.6%)	92 (24.3%)	107 (28.4%)	117 (32.3%)
Lipid Rx, n (%)	165 (22.3%)	53 (14.2%)	112 (30.6%)	95 (26.3%)	70 (18.5%)	67 (17.8%)	98 (27.1%)
Prevalent CVD, n (%)	89 (12.0%)	23 (6.2%)	66 (18.0%)	60 (16.6%)	29 (7.7%)	38 (10.1%)	51 (14.1%)
Prevalent AF, n (%)	27 (3.7%)	4 (1.1%)	23 (6.3%)	19 (5.3%)	8 (2.1%)	12 (3.2%)	15 (4.1%)
Prevalent Cancer, n (%)	110 (14.9%)	40 (10.7%)	70 (19.1%)	53 (14.7%)	57 (15.1%)	53 (14.1%)	57 (15.7%)
Prevalent Stroke, n (%)	10 (1.4%)	3 (0.8%)	7 (1.9%)	5 (1.4%)	5 (1.3%)	4 (1.1%)	6 (1.7%)

Tab s5 and Tab s6 (Online Resource 1) show the summary statistics for the EAA and PoA metrics in the full sample and each of the age, sex, and CMV strata in the immune sample and protein subsample, respectively. There was no notable difference in DNAm aging summary statistics between the immune sample and protein subsample. The average EAA and PoA levels between strata for all six DNAm aging outcomes are significantly (*p* < 0.05) higher among men, indicating that the males exhibited less healthy DNAm aging compared to females by all measures. Only the PoA.DunedinPACE measures significantly (*p* < 0.05) differed between the age groups, with the older stratum having a larger PoA. The first generation DNAm aging metrics EAA.PCHorvath1, EAA.PCHorvath2, and EAA.PCHannum measured at Exam 8 was significantly (*p* < 0.05) higher in participants who were CMV positive at Exam 7. A Pearson correlation plot for the six DNAm aging measures in the full immune cell phenotype sample is shown in Fig s3 (Online Resource 2). The EAA metrics from the first-generation clocks are all strongly correlated with each other (0.76 < *r* < 0.85), but between the second-generation clocks, this correlation was only moderate (*r* = 0.50). Interestingly, EAA.PCPhenoAge and EAA.PCHannum are quite strongly correlated (*r* = 0.83) and the PoA.DunedinPACE was moderately correlated with the EAA.PCGrimAge (*r* = 0.62).

### Overall summary

Complete regression results for analyses of all immune cell phenotypes with all DNAm aging outcomes for Model 1 and Model 2 are provided in Tab s1 (Online Resource 3). Regression results for analyses of all OLINK proteins with all DNAm aging outcomes for Model 1 and Model 2 are provided in Tab s2 (Online Resource 3).

**Fig. 2:**
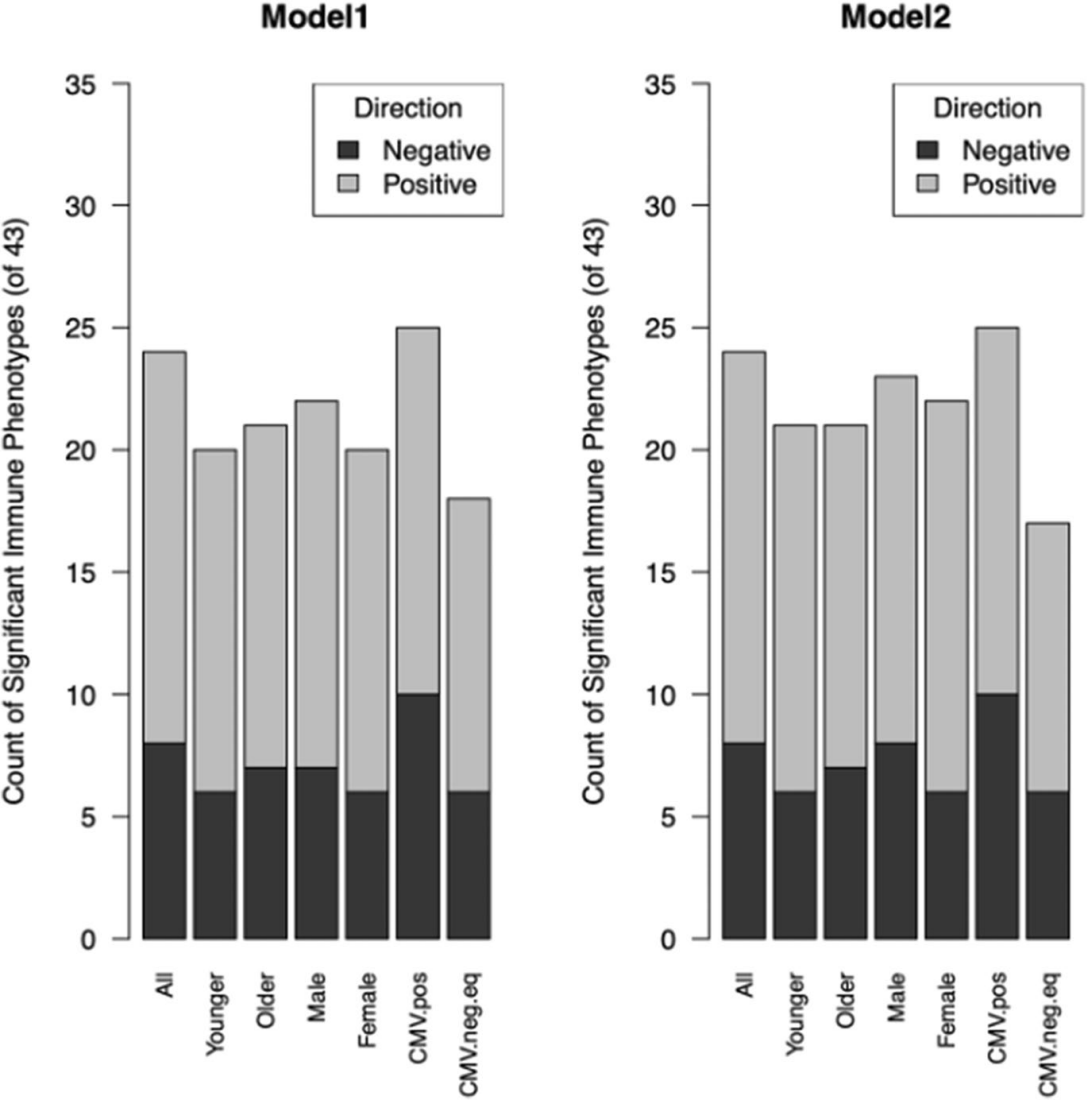
Stacked bar plot showing counts and direction of effect of FDR-significant immune cell phenotypes in the strata

**Fig. 3:**
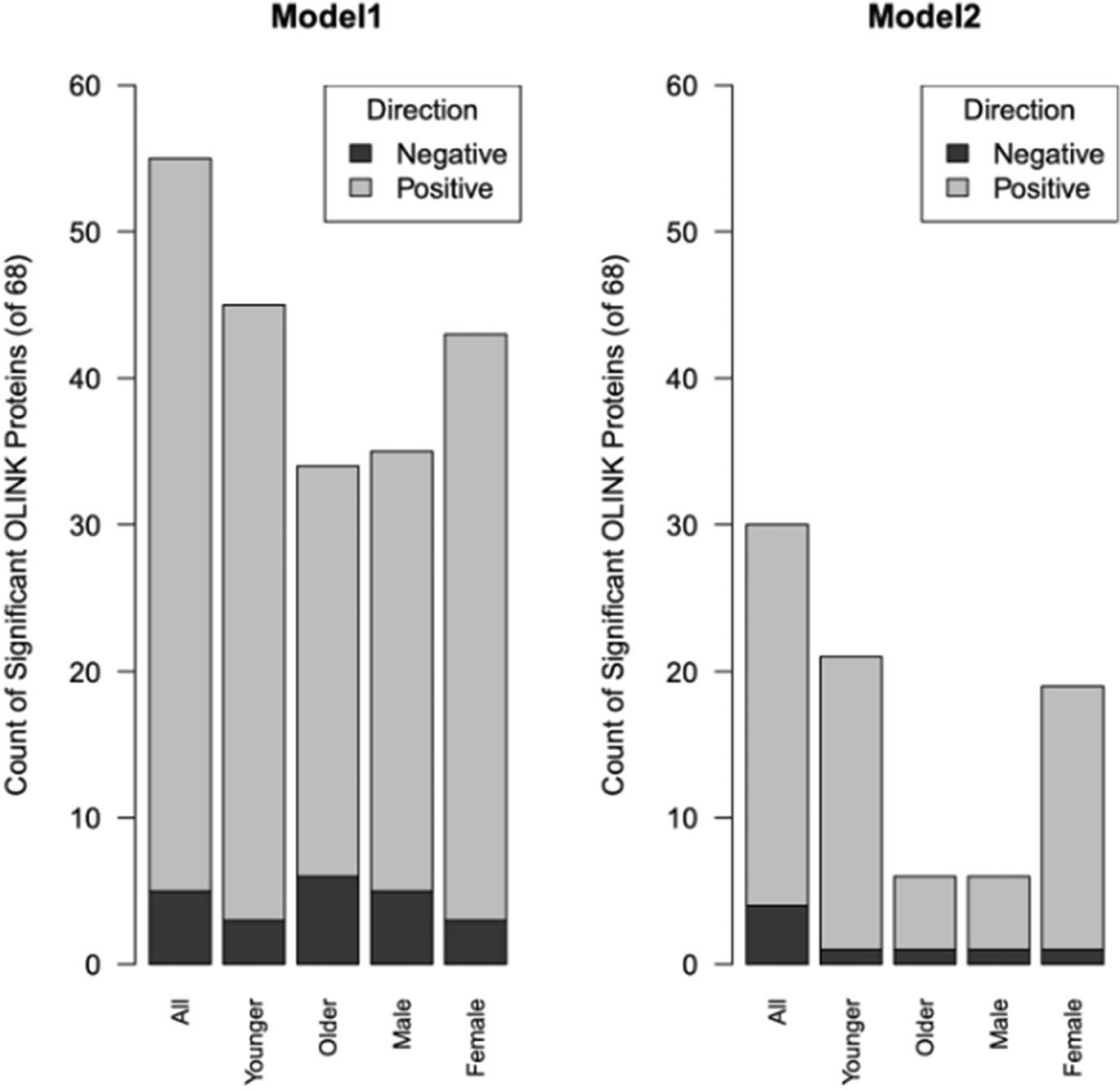
Stacked bar plot showing counts and direction of effect of FDR-significant OLINK proteins in the strata

Figures 2 and 3 are stacked bar plots showing the number of unique immune cell phenotypes and inflammatory proteins that are significantly (FDR < 0.05) associated with at least one of the six DNAm aging outcomes in the full sample and each stratum, adjusting for Model 1 and Model 2 covariates. A large proportion of the immune cell phenotypes and inflammatory proteins exhibit significant prospective association with the DNAm aging outcomes after an average of 6 years of follow-up: 24 of the 43 immune cell phenotypes and 55 of the 68 inflammatory proteins investigated were significantly associated with at least one of the six DNAm aging metrics after controlling for the basic Model 1 covariates. More of the significant associations identified in the immune cell and protein analyses were in the positive direction (higher levels of the predictor were associated with higher EAA or POA (unhealthy aging). For the immune cells, 8 of 24 associations were negative, while in the protein analyses, only 5 of the 55 associations were in the negative direction. The direction of association for each significant predictor was consistent across the different DNAm aging outcomes (e.g., if a protein was negatively associated with one DNAm aging outcome, it was negatively associated with the others as well). Tab s3 and Tab s4 (Online Resource 3) summarize the unique immune cell phenotypes and proteins that have positive and negative associations with at least one of the six DNAm aging outcomes.

After adjusting for prevalent diseases and individual CVD risk factors (Model 2), the counts of the unique and significant immune cell phenotypes remained much the same (Fig. 2). However, there were notably fewer significant proteins associated with the DNAm aging outcomes across the strata under Model 2 compared to Model 1 (Fig. 3). In the full sample, the number of inflammatory proteins having a significant association with at least one of the DNAm aging outcomes decreased to 30 from 55 for Model 1.

In the stratified analyses, we observe that under both Model 1 and Model 2, there is a slightly larger number of significant immune cell phenotypes among the CMV-positive stratum compared to the CMV-negative and equivocal stratum (Fig. 2). For both Model 1 and Model 2, the number of significant proteins was notably larger in the younger group compared to the older group. Additionally, for Model 2, a greater number of proteins were associated with the DNAm aging outcomes among females compared to males (Fig. 3).

**Fig. 4:**
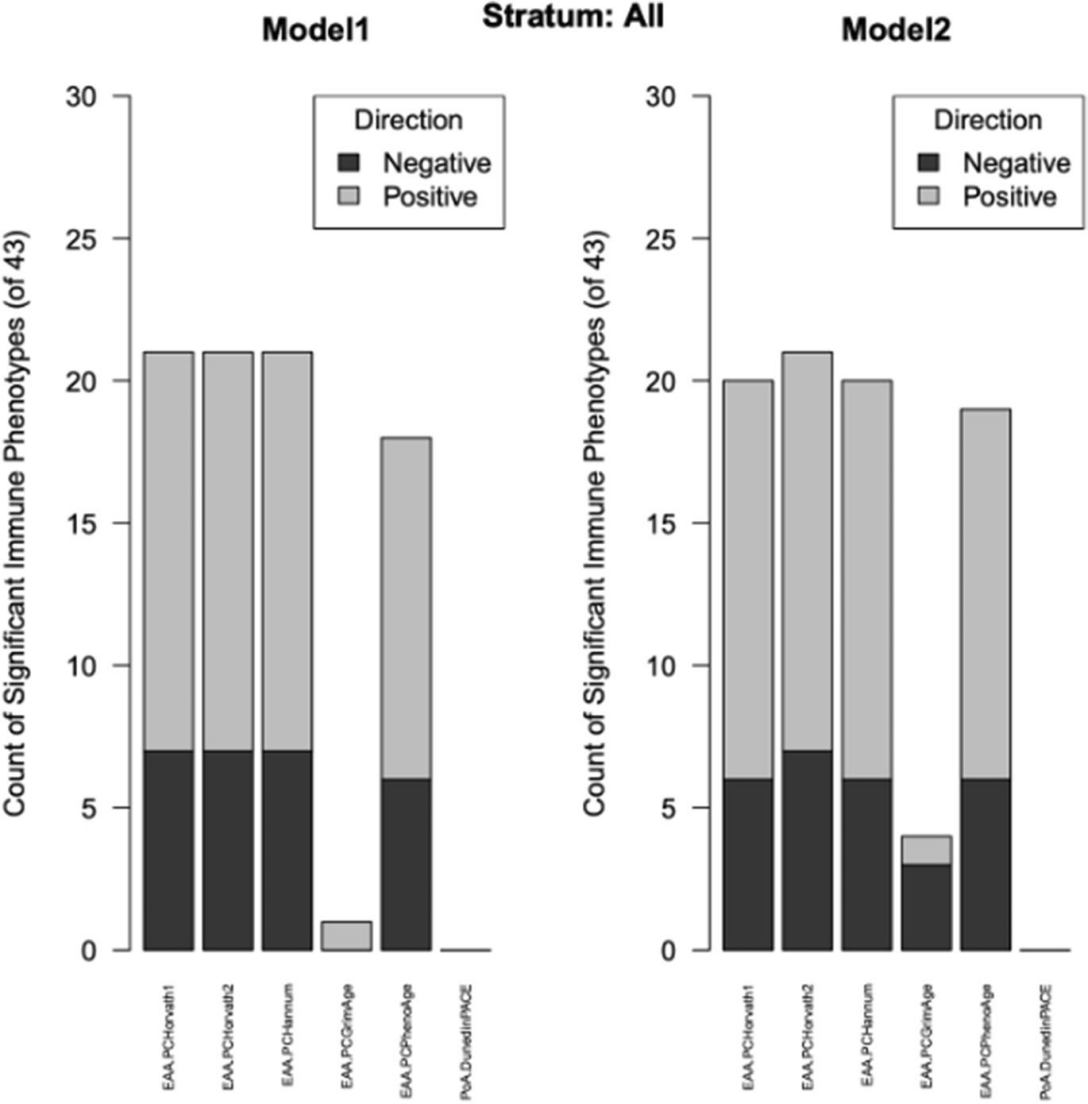
Stacked bar plot showing counts and directionality of immune cell phenotypes significantly associated with each DNAm aging outcome in the full sample

**Fig. 5:**
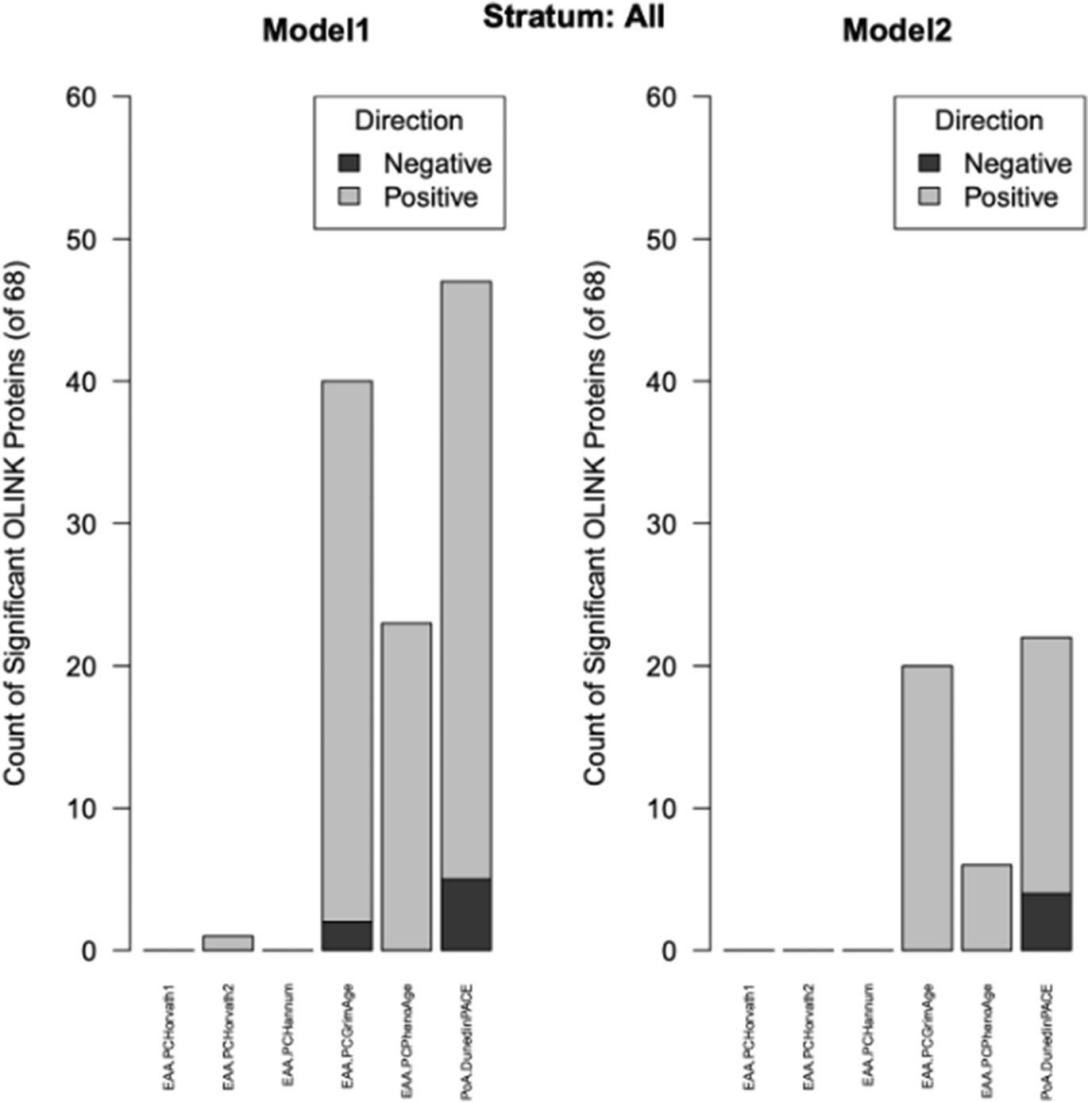
Stacked bar plot showing counts and directionality of OLINK proteins significantly associated with each DNAm aging outcome in the full sample

Figures 4 and 5 show the number of unique immune cell phenotypes and inflammatory proteins that are significantly (FDR < 0.05) associated with each of the six DNAm aging outcomes in the full sample, adjusting for Model 1 and Model 2 covariates. Complete counts and descriptions of exactly which immune cell phenotypes and proteins are associated with each of the DNAm aging metrics and their direction of association for all strata are provided in Tab s5 and Tab s6 (Online Resource 3). The immune cell phenotypes had the greatest number of associations with the three first-generation clock metrics and the second-generation metric EAA.PCPhenoAge, had very few associations with the EAA.PCGrimAge metric, and had no associations with the PoA.DunedinPACE metric (Fig. 4). The inflammatory proteins were primarily associated with the second- and third-generation clock metrics EAA.GrimAge, EAA.PhenoAge, and PoA.DunedinPACE and had very few or no associations with metrics from the first-generation PC clocks (Fig. 5). EAA.PCPhenoAge was the only outcome that had a notable number of significant associations with both the immune cell phenotypes and inflammatory proteins. These patterns extended to all strata.

### Association of DNAm aging outcomes with immune cell phenotypes in the full sample

**Fig. 6:**
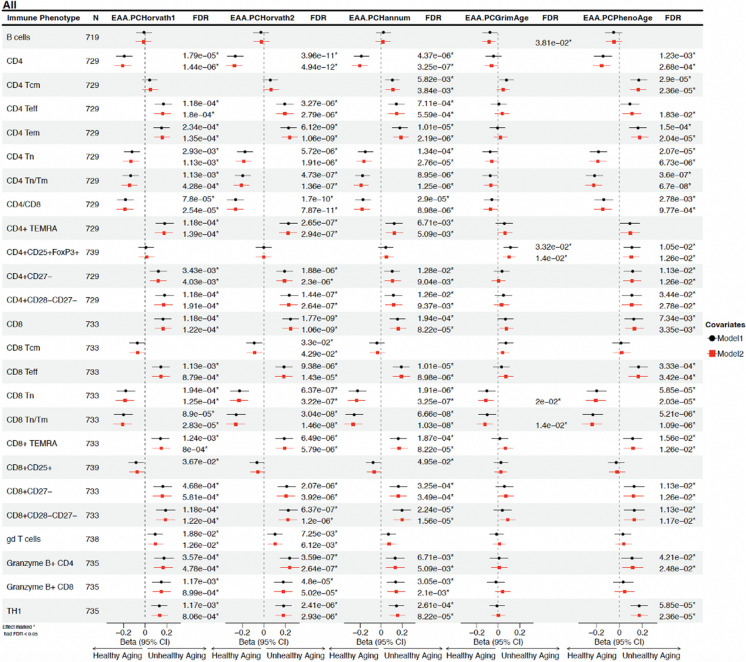
Forest plot of effect sizes and 95% confidence intervals for significant (FDRs < 0.05 noted) prospective associations between immune cell phenotypes and select DNAm aging outcomes in the full sample using Model 1 and Model 2 covariates

Figure 6 provides a visual overview of the effects of the immune cell phenotypes that have significant associations with the DNAm aging metrics in the full sample after adjustment for Model 1 and Model 2 covariates. FDRs < 0.05 are noted; absence of an FDR indicates a non-significant association. PoA.DunedinPACE has no significant associations with the immune cell phenotypes and was therefore not included in Fig. 6.

Descriptions of the set of significant immune cell phenotypes in each strata for each model, their directionality, and the number and description of which DNAm aging outcomes they are associated with can be found in Tab s7 (Online Resource 3).

We identified 8 immune cell phenotypes significantly negatively associated with DNAm aging metrics in the full sample, adjusting for Model 1 covariates. In order of most frequent (i.e., significantly associated with most DNAm aging outcomes) to least frequent, these phenotypes are CD8 Tn/Tm, CD8 Tn, CD4 Tn/Tm, CD4 Tn, CD4, CD4/CD8, CD8 + CD25 +, and CD8 Tcm. Higher levels of these T cell subtypes and larger ratios at baseline are associated with lower prospective EAA measures and therefore healthier DNAm aging about 6 years later. As shown in Fig. 6, the strongest negative associations between these immune cell phenotypes and the DNAm aging metrics include CD4 and CD4/CD8 with EAA.PCHorvath2 (for both, beta = − 0.26, 95% CI = [− 0.34, − 0.19]) and CD8 Tn/Tm with EAA.PCHannum (beta = − 0.26, 95% CI = [− 0.34, − 0.18]).

Under Model 1, we also identified 16 immune cell phenotypes significantly positively associated with DNAm aging in the full sample. In order of most frequent to least frequent, these phenotypes are CD8 + CD28 − CD27 −, CD8 Teff, CD8 + TEMRA, CD4 Tem, TH1, Granzyme B + CD4, CD8 + CD27 −, CD4 + CD28 − CD27 −, Granzyme B + CD8, CD8, CD4 + CD27 −, CD4 + TEMRA, CD4 Teff, CD4 Tcm, CD4 + CD25 + FoxP3 +, and gd T cells. Higher levels of these T cell subtypes at baseline are associated with higher prospective EAA measures and less healthy DNAm aging about 6 years later. As shown in Fig. 6, the strongest associations of these immune cell phenotypes with the DNAm aging metrics include CD8 (beta = 0.25, 95% CI = [0.17, 0.32]), Granzyme B + CD4 (beta = 0.24, 95% CI = 0.15 to 0.33), and CD4 + CD28 − CD27 − (beta = 0.24, 95% CI = [0.15, 0.32]) with the EAA.PCHorvath2 outcome.

After adjusting for prevalent diseases and CVD risk factors (Model 2), the directions of effect for the immune cell phenotype associations remain the same. Most associations for the full sample had approximately the same, if not slightly larger, effect size and similar or slightly smaller *p*-value. Thus, as we observe in Fig. 6, some associations in the full sample that were non-significant under Model 1 become significant under Model 2. These findings also extend to the age, sex, and CMV strata. Of particular note, after adjusting for prevalent disease and CVD risk factors, B cells became significant while CD8 + CD25 + T cells became insignificant in the full sample.

### Stratified immune phenotype analyses

Fig s4 and Fig s5 (Online Resource 2) are forest plots for the prospective associations between the immune cell phenotypes and all six DNAm aging outcomes in the full sample and all strata using the two different covariate models for the 27 immune cell phenotypes with significant associations with one of the outcomes in either the full sample or at least one of the strata. The complete results for all immune cells and strata are in Tab s1, Online Resource 3.

We did not formally test for the significance of the difference between stratified effects. Our determination of a “notable” difference was loosely based on the 95% CI of one stratum not overlapping with the point estimate of the other stratum. Tab s8 (Online Resource 3) details associations with notable differences between strata, if the effect was significant in at least one of the strata (we will only explicitly mention associations for which this is true), if the effect was significant in both of the strata, we indicate which stratum had the stronger effect.

Nearly all of the significant immune cell phenotype associations observed in the sex, age, and CMV stratified analyses were also significant in the full sample analysis. Under Model 1, the effect sizes for the significant associations between the immune cell phenotypes and the DNAm aging outcomes did not appear to notably differ by age and sex, and when they did, they often only differed with respect to a single DNAm aging metric. Some exceptions that had notable age differences across multiple DNAm aging outcomes included the negative association between CD4/CD8 and EAA from all three first-generation PC clocks (stronger in older participants than younger), which is likely driven by the positive association of CD8 with the EAA from these three first-generation PC clocks (also stronger in older participants than younger participants). Notable sex differences across multiple DNAm aging outcomes included the positive association of CD4 Teff with EAA.PCHorvath1 and EAA.PCPhenoAge (stronger in females than males) and the negative association of CD8 Tcm with EAA from the two PCHorvath clocks (EAA.PCHorvath1, EAA.PCHorvath2) (stronger in males than females).

A larger number of CMV stratum effect sizes differed compared to sex and age strata. Notable CMV differences across multiple DNAm aging outcomes included the negative association of CD8 Tcm with the EAA from all three first-generation PC clocks; the positive associations of CD8 + CD27 −, CD8 + CD28 − CD27 −, and Granzyme B + CD4 with EAA from all three first-generation PC clocks; and the positive associations of CD8 Teff and Granzyme B + CD8 with EAA from the two PCHorvath clocks. The CMV-positive group typically had a larger effect size compared to the CMV-negative/equivocal stratum and the associations were FDR-significant in the CMV-positive group, but not the CMV-negative group. This explains the larger number of associations in the CMV-positive group observed in Fig. 2. The additional covariates in Model 2 did not substantively affect the stratified findings.

### Association of DNAm aging outcomes with inflammatory proteins in the full sample

**Fig. 7:**
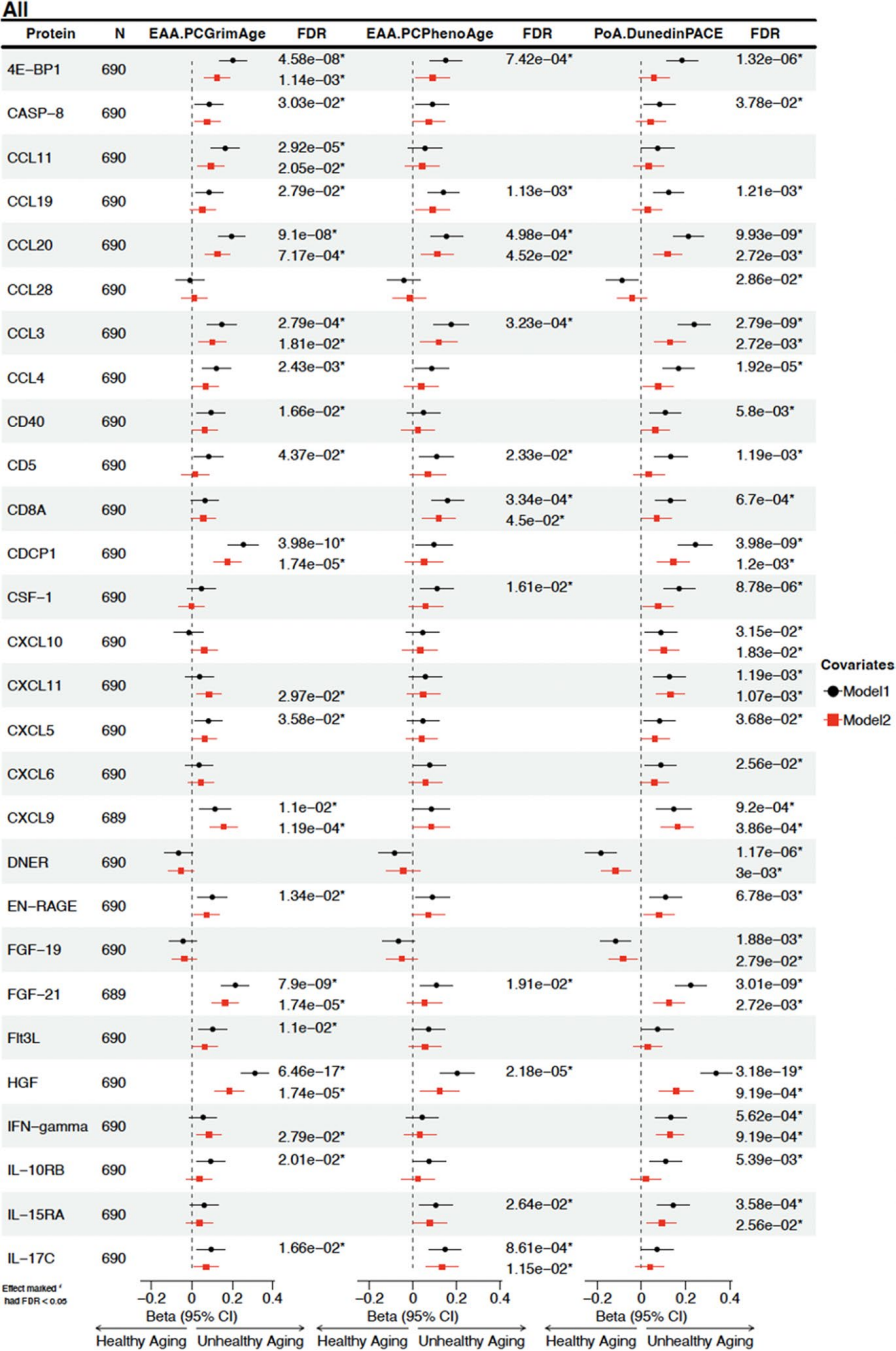

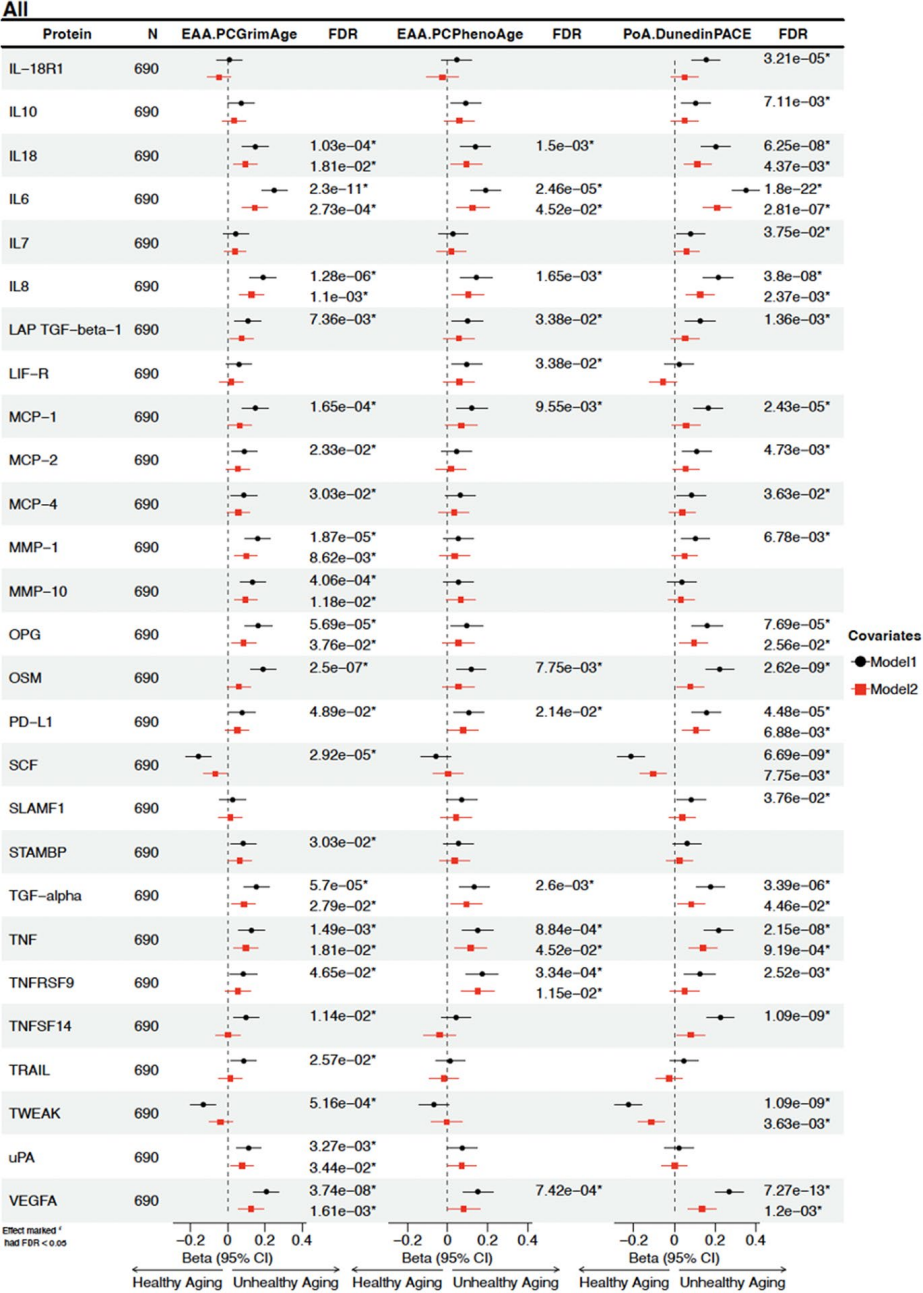
Forest plot of effect sizes and 95% confidence intervals for significant (FDRs < 0.05 noted) prospective associations between inflammatory protein biomarkers and select DNAm aging outcomes in the full sample using Model 1 and Model 2 covariates

The significant associations between the inflammatory protein biomarkers and selected DNAm aging metrics identified in the full sample adjusting for Model 1 and Model 2 covariates are shown in Fig. 7 (parts a and b). FDRs < 0.05 are provided. EAA.PCHorvath1 and EAA.PCHannum had no significant associations with the inflammatory proteins, and only CD8A was significantly associated with EAA.PCHorvath2 under Model 1 (beta = 0.14, 95% CI = [0.07, 0.22]). These DNAm metrics are therefore not included in Fig. 7. The effect sizes (all reported in SD units of predictor and outcome) of the proteins with PoA.DunedinPACE tended to be larger than the effect sizes for the EAA.PCPhenoAge and EAA.PCGrimAge metrics.

Descriptions of the set of significant proteins in each strata for each model, their directionality, and the number and description of which DNAm aging outcomes they are associated with can be found in Tab s9 (Online Resource 3).

Five inflammatory proteins are negatively associated with at least one of the DNAm aging metrics (i.e., with healthier aging) in the full sample adjusting for Model 1 covariates. TWEAK (aka. TNFSF12) and SCF are significantly negatively associated with both EAA.PCGrimAge and PoA.DunedinPACE. DNER, FGF-19, and CCL28 are negatively associated with PoA.DunedinPACE. Higher levels of these inflammatory proteins at baseline are associated with lower prospective PoA.DunedinPACE or EAA.PCGrimAge measures about 6 years later. The effects of these five inflammatory proteins on PoA.DunedinPACE range in size from − 0.23 to − 0.09 SD units per SD unit increase in protein level, with the coefficients for TWEAK (beta = − 0.23, 95% CI = [− 0.29, − 0.16]) and SCF (beta = − 0.21, 95% CI = [− 0.28, − 0.15]) being the largest.

Under Model 1, we also identified 50 unique inflammatory proteins associated with future unhealthy aging. Forty-two of these are associated with PoA.DunedinPACE (alone or in addition to EAA.PCPhenoAge and/or EAA.PCGrimAge), and the remaining eight are associated with only EAA.PCPhenoAge and/or EAA.PCGrimAge; all appear in Fig. 7. Nineteen proteins are associated with all three of the second- and third-generation metrics: 4E-BP1, CCL19, CCL20, CCL3, CD5, CD8A, FGF-21, HGF, IL18, IL6, IL8, LAP TGF-beta-1, MCP-1, OSM, PD-L1, TGF-alpha, TNF, TNFRSF9, and VEGFA. Higher levels of these inflammatory proteins at baseline are associated with higher prospective PoA and EAA and thus less healthy DNAm aging about 6 years later. As we observe in Fig. 7, positive effect sizes for PoA.DunedinPACE range from 0.08 to 0.35 SD units per unit increase in protein level, with the coefficients for IL6 (beta = 0.35, 95% CI = [0.28, 0.42]) and HGF (beta = 0.34, 95% CI = [0.27, 0.41]) being the largest. The strongest positive associations between these inflammatory proteins and EAA.PCGrimAge were for HGF (beta = 0.31, 95% CI = [0.24, 0.38]), CDCP1 (beta = 0.26, 95% CI = [0.18, 0.33]), and IL6 (beta = 0.25, 95% CI = [0.18, 0.32]). The protein effect sizes tended to be smaller and less significant for EEA.PCPhenoAge than the other two metrics.

In contrast to the immune cell associations, after additionally adjusting for prevalent diseases and CVD risk factors (Model 2), the direction of effects for the inflammatory proteins remains the same, but the associations generally have attenuated effect sizes and lower significance. Half or fewer of the proteins associated using Model 1 reach FDR < 0.05 significance after adjusting for the CVD risk factors for each of the three second- and third-generation metrics, and only 3 of the 19 proteins associated with all 3 metrics remain significant for all 3 outcomes: CCL20, IL6, and TNF.

### Stratified protein analyses

Figs. s6 and s7 (Online Resource 2) are forest plots for the prospective associations between 56 inflammatory protein biomarkers and all six DNAm aging outcomes in the full sample and all strata using the two different covariate models. In these stratified analyses, we limit the associations we look at to those that are significant in either the full sample or one of the strata. All association results for all strata are presented in Online Resource 3 Tab s2.

As for the immune cell analyses, we did not formally test for differences between strata effect sizes for the proteins and used the same criterion to note potentially interesting (“notable”) differences (Tab s10, Online Resource 3). All but one of the significant protein associations observed in the sex- and age-stratified analyses were also significant in the full sample analysis. Under Model 1, most of the effect sizes were similar in males and females. Some exceptions noted across multiple DNAm aging outcomes include the positive associations of FGF-21 and IL10 with EAA.PCGrimAge, EAA.PCPhenoAge, and PoA.DunedinPACE (stronger in females than males), as well as the positive association of VEGFA with EAA.PCPhenoAge and PoA.DunedinPACE (also stronger in females than males).

In the age-stratified analyses under Model 1, we found that the effects of several of the significant inflammatory proteins on future EAA and PoA measures differed between the younger and older strata. Notable age differences across multiple DNAm aging outcomes included the positive associations of 4E-BP1, IL18, and OPG with EAA from the two second-generation PC clocks (EAA.PCGrimAge, EAA.PCPhenoAge); the positive associations of CDCP1, Flt3L, HGF, and IL6 with EAA.PCPhenoAge and PoA.DunedinPACE; the positive association of TNF and EAA.PCGrimAge and PoA.DunedinPACE; and the negative associations of CX3CL1 and SCF with EAA.PCGrimAge and PoA.DunedinPACE. For all of these, the younger participants consistently had stronger effect sizes compared to older participants, with associations in the younger stratum being FDR significant but not in the older participants.

Because all effect sizes for the inflammatory proteins are attenuated after controlling for CVD risk factors, most associations in the full sample and strata become non-significant under Model 2. The only association that maintains a notable difference between stratum effects across multiple DNAm aging metrics after adjusting for CVD risk is that of Flt3L with EAA.PCGrimAge and PoA.DunedinPACE.

## Discussion

Our study expands the understanding of the relationship between immune cells and inflammatory protein biomarkers and DNAm aging metrics by considering larger panels of immune cells and proteins, by using newer, more reliable PC-based DNAm aging metrics and the Pace of Aging metric [13], and by testing for association between the proteins or immune cells and subsequent DNAm aging metrics measured an average of 6 years later. We found that a large proportion of the 43 immune cell phenotypes and 68 inflammatory proteins had prospective associations with some of the six PC-based DNAm aging. The associations with the improved PC versions of the most widely used epigenetic clocks in research suggest that immune cell phenotypes and inflammatory biomarkers contribute to whether one experiences healthy biological aging later in life. All of our analyses were adjusted for age at immune cell or protein measurement, so that the reported significant associations are independent of age.

### Immune cells and EAA measures

The immune cell phenotypes were associated primarily with DNAm aging metrics based on first-generation PC Horvath and Hannum clocks that were not adjusted for cell types, and the second-generation PC PhenoAge, and their effects were not altered by the inclusion of cardiovascular covariates. Prior work imputed immune cell abundance based on DNA methylation data, whereas our study directly measured the immune cells using flow cytometry, and we further extend the work by reporting the association between immune cells and DNAm aging metrics measured on average 6 years later.

Prior investigations that developed and tested various DNAm aging measures, including the PC-based measures investigated in this study, reported cross-sectional associations with a variety of immune cells [10–12, 14, 15] including negative correlations with naïve CD4 + and CD8 + T cells and positive correlations with exhausted CD8 + T cells, consistent with known age-related changes in the immune system. However, despite previous work showing negative B cell [10, 11] and NK cell [11] and positive monocyte [10, 11] cross-sectional correlations with DNAm aging, our work suggests that this relationship may not extend longitudinally across a 6-year timespan. Aging-related changes in the immune system are characterized by changes in T-cell subsets, including reduction in naïve T cells (Tn), expansion of memory and effector T cells, and T cell senescence [34, 35]. Prior work from the Health and Retirement Study demonstrated strong cross-sectional associations between specific T cell naïve and memory subsets and derived ARIP ratios and a measure of biologic age derived from biomarkers representing physiologic functions [21]. In that study, CD8 Tn and CD8 Tn/Tm were observed to have the strongest association with biologic age; however, after adjusting for chronologic age, sex, race/ethnicity, and CMV (similar to our model 1), only CD4 Tn and CD4 Tn/Tm remained significantly negatively associated. Our work focused on epigenetic measures of biological aging and identified several immune cell phenotypes negatively associated with first-generation and PhenoAge EAA metrics measured about 6 years later. Higher proportions of immune cell phenotypes and larger ARIP ratios were observed to be associated with healthier future DNAm aging (lower EAA) specifically higher levels of CD4 and CD8 Tn cells and larger CD4 and CD8 Tn/Tm ratios (naïve to memory cell ratio), and the CD4/CD8 ratio. Changes in naïve-memory balance and T cell senescence are hallmarks of T cell aging; higher levels of naïve cells and ARIPs may reflect better immune aging [34], and hence, we hypothesize are the immune cell metrics more likely to be associated with more favorable biologic aging. We also identified a number of immune cell phenotypes with positive prospective associations with EAA defined from the first generation and PhenoAge clocks. Higher proportions of both CD4 and CD8 memory, effector, and TEMRA cells as well as immune cells representing replicative senescence affect the epigenetic aging clocks 6 years in the future, portending poorer biologic aging. In our cross-sectional study of immune cells with age, sex, and clinical covariates [20], we tested for the association between age at Exam 7 and the immune cells as outcomes. Among the immune phenotypes that had a negative association with the first generation and/or PhenoAge clocks in our current work, four also had a cross-sectional negative association with age: CD4 + Naïve, CD8 + Naïve, CD4 + Tn/Tm, and CD8 + Tn/Tm. Among the immune phenotypes that had a positive association with the first generation and/or PhenoAge clocks, four also had a cross-sectional positive association with age: CD8 + CD27 −, CD8 + CD28 − CD27 −, CD8 Teff, and CD8 + Granzyme B +. The other immune cells associated with biologic age were not associated cross-sectionally with age. Hence, our observations support the hypothesis that better immune aging (preservation of higher naïve T cells and composite age-related immune phenotypes) associates with slower biologic age measured with epigenetic clocks while more exhausted/senescent type immune phenotypes associate with faster biologic age.

Many of the CMV-positive stratum association effect sizes were larger than those in the CMV-negative/equivocal stratum, and these associations were more often in the positive direction, suggesting CMV seropositivity could worsen the effect of harmful immune cell phenotypes on future DNAm aging. CMV infection is common among older adults and is known to associate with profound alterations on the immune system with expansion of memory, effector, and TEMRA cells [22, 36, 37]. Therefore, it is not surprising that we observed stronger immune cell phenotype effect sizes in the CMV-positive stratum compared to CMV-negative or equivocal participants.

### Inflammatory markers and EAA measures

The inflammatory proteins were associated primarily with the PC-based second-generation GrimAge and third-generation DunedinPACE metrics, and to a lesser extent with the PhenoAge metric. For many of the proteins, the effect sizes were larger in the younger (age < 60) stratum, suggesting that inflammation at a younger age could have a more detrimental effect on subsequent DNAm aging health compared to inflammation at an older age. Most of the 68 inflammatory proteins were positively associated with PhenoAge and GrimAge EAA and DunedinPACE PoA, an average of 6 years later, and 19 proteins were associated with all three of these DNAm measures. Only five of the inflammatory proteins, TWEAK, SCF, DNER, CCL28, and FGF-19, appear to play a protective role in DNAm aging, with higher protein levels associated with a reduced rate of DNAm aging ~ 6 years later for the PoA metric. These five proteins appear to have more tissue-specific roles in inflammation, compared to the 19 proteins that are associated with accelerated DNAm age for all three metrics. All effects were diminished substantially after including cardiovascular covariates in the model. This may suggest that biologic pathways related to CVD play a confounding or mediating role in the prospective association between baseline inflammation and EAA or PoA. Both CVD and individual risk factors, including BMI and smoking, as well as the composite FHS CVD risk score, associate with epigenetic age acceleration, establishing the confounder/mediator-outcome relationship at least cross-sectionally [38]. Additionally, systemic inflammation has been shown to mediate the association between subclinical atherosclerosis and EAA [39].

Our findings of large numbers of associations between inflammatory proteins and DNAm age metrics appear to contradict the conclusion made by Cribb et al. [6], who found little evidence of longitudinal associations between inflammatory markers and epigenetic aging across 10 + years of follow-up. The discrepancy may be explained by differences between our study and Cribb et al. First, we use a larger number of inflammatory biomarkers, with only five markers in common (IL6, IL8, IL10, IFN-gamma, and TNF). Second, Cribb et al. controlled for baseline EAA, and thus, their associations are conditional on baseline aging health. Third, by minimizing technical noise, the newer PC-based clocks used in our study may have improved the power to detect associations. Finally, the association between inflammation and DNAm aging measures may be stronger after a shorter follow-up period (in our case, 6 years) and become weaker with increasing follow-up time.

We observed that the immune cell phenotypes and inflammatory proteins seemed to be associated with different types of epigenetic clocks: the immune cell phenotypes tended to be associated with DNAm aging metrics derived from the first-generation PC clocks, while the inflammatory proteins tended to be associated with DNAm aging metrics derived from the second-generation PC clocks and the third-generation PoA clock. The first-generation epigenetic clocks we used predict chronological age based on DNAm and do not adjust out the effects of blood cell counts in their calculation of EAA. Changes in immune cell populations with respect to chronological age are well documented, so a connection between immune cell composition and DNAm-estimated chronological age and EAA metrics seems plausible. The second-generation PhenoAge clock was built to include immune phenotypic aging measures such as lymphocyte percent and white blood cell count. Consequently, it has been shown that PhenoAge’s EAA measure captures aspects of immunosenescence in blood. The three second- and third-generation epigenetic clocks—PCPhenoAge, PCGrimAge, and DunedinPACE—were developed using algorithms that incorporated inflammatory proteins (mainly CRP) as some of their inputs [10, 11, 13]. Thus, it seems reasonable that other inflammatory proteins would be associated with these DNAm aging metrics. Thus, these clocks may be better equipped to capture systemic inflammaging compared to first-generation epigenetic clocks, which do not incorporate inflammatory biomarkers of aging, thus potentially explaining the lack of significant associations observed between the proteins and the first-generation epigenetic clocks.

Several limitations to this study are worth noting. First, the FHS Offspring participants are predominantly white and of European ancestry and are therefore not representative of the diversity of the U.S. population. Second, the modest sample size limits the statistical power of our analyses, particularly in the stratified analyses. Third, the immune cell phenotyping, inflammatory marker profiling, and DNAm measurements were collected only once in the FHS, so our analyses and conclusions are limited by data availability. We were unable to adjust for baseline EAA and PoA at Exam 7, and the follow-up period of approximately 6 years is too short to make predictions about epigenetic aging farther into the future. Future studies may benefit from longitudinal immune phenotype, inflammatory biomarker, and DNAm measurements, which would allow for the adjustment for baseline DNAm aging health status and a more in-depth assessment of temporal patterns in the relationship between immune cell phenotypes, proteins, and DNAm aging outcomes. Fourth, due to the observational design of this study and the limited data available, the causation of epigenetic aging cannot be definitively attributed to immune cell phenotypes and inflammatory proteins. Elucidation of cause-and-effect relationships will require future longitudinal cohort studies and mechanistic evaluations. Finally, because the immune cells were measured at a time point ~ 6 years prior to the DNAm measurements, we can only study how immune cells relate to later biologic aging, but cannot study how the biologic clocks affect immune aging.

Despite these limitations, to our knowledge, this study is one of the first attempts to investigate prospective relationships between baseline immune cell composition measured via flow cytometry and inflammatory protein levels and DNAm age measured several years later. Further, since we used a large immune and inflammatory marker panel and multiple generations of epigenetic clocks, this work presents one of the most comprehensive mappings of immune and inflammatory contributions to EAA and PoA to date. Because of the more recent development of PC-based first- and second-generation epigenetic clocks, this study is one of the few to use EAA from PC-trained clocks. Finally, the extensive characterization of the FHS participants permitted us to observe the residual effects of the protein levels after accounting for CVD risk factors, extending our understanding of potential mediating factors in the relationships between inflammation and biologic age.

## Conclusions

In conclusion, many circulating immune cell phenotypes and inflammatory protein biomarkers have been shown to prospectively associate with epigenetic aging health across an approximate 6-year observation period, with most of the investigated markers being associated with poorer biological aging. Stratified analyses suggest possible differences in immune effects between CMV-positive and CMV-negative or equivocal participants and differences in protein effects between younger and older participants. We found that most of the immune associations were with first-generation PC clocks, while most of the protein associations were with PC-trained second- and third-generation clocks.

## Supplementary information

Below is the link to the electronic supplementary material.

ESM1 (XLSX 51.5 KB)

ESM2 (PDF 716 KB)

ESM3 (XLSX 825 KB)

## Data Availability

Framingham Heart Study Offspring data are available for request via the NHLBI Biological Specimen and Data Repositories Information Coordinating Center (BioLINCC).

## References

[1] Niccoli T, Partridge L. Ageing as a risk factor for disease. Curr Biol. 2012;22:R741–52. 10.1016/j.cub.2012.07.024. 22975005 10.1016/j.cub.2012.07.024

[2] Moqri M, Herzog C, Poganik JR, et al. Biomarkers of aging for the identification and evaluation of longevity interventions. Cell. 2023;186:3758–75. 10.1016/j.cell.2023.08.003. 37657418 10.1016/j.cell.2023.08.003 PMC11088934

[3] Ferrucci L, Gonzalez-Freire M, Fabbri E, et al. Measuring biological aging in humans: a quest. Aging Cell. 2020;19:e13080. 10.1111/acel.13080. 31833194 10.1111/acel.13080 PMC6996955

[4] López-Otín C, Blasco MA, Partridge L, et al. Hallmarks of aging: an expanding universe. Cell. 2023;186:243–78. 10.1016/j.cell.2022.11.001. 36599349 10.1016/j.cell.2022.11.001

[5] Aiello A, Farzaneh F, Candore G, et al. Immunosenescence and its hallmarks: how to oppose aging strategically? A review of potential options for therapeutic intervention. Front Immunol. 2019;10:2247. 10.3389/fimmu.2019.02247. 31608061 10.3389/fimmu.2019.02247 PMC6773825

[6] Cribb L, Hodge AM, Yu C, et al. Inflammation and epigenetic aging are largely independent markers of biological aging and mortality. The Journals of Gerontology: Series A. 2022;77:2378–86. 10.1093/gerona/glac147. 10.1093/gerona/glac147 PMC9799220 35926479

[7] Horvath S. DNA methylation age of human tissues and cell types. Genome Biol. 2013;14:3156. 10.1186/gb-2013-14-10-r115. 10.1186/gb-2013-14-10-r115 PMC4015143 24138928

[8] Horvath S, Oshima J, Martin GM, et al. Epigenetic clock for skin and blood cells applied to Hutchinson Gilford Progeria Syndrome and ex vivo studies. Aging. 2018;10:1758–75. 10.18632/aging.101508. 30048243 10.18632/aging.101508 PMC6075434

[9] Hannum G, Guinney J, Zhao L, et al. Genome-wide methylation profiles reveal quantitative views of human aging rates. Mol Cell. 2013;49:359–67. 10.1016/j.molcel.2012.10.016. 23177740 10.1016/j.molcel.2012.10.016 PMC3780611

[10] Levine ME, Lu AT, Quach A, et al. An epigenetic biomarker of aging for lifespan and healthspan. Aging. 2018;10:573–91. 10.18632/aging.101414. 29676998 10.18632/aging.101414 PMC5940111

[11] Lu AT, Quach A, Wilson JG, et al. DNA methylation grimage strongly predicts lifespan and healthspan. Aging. 2019;11:303–27. 10.18632/aging.101684. 30669119 10.18632/aging.101684 PMC6366976

[12] Higgins-Chen AT, Thrush KL, Wang Y, et al. A computational solution for bolstering reliability of epigenetic clocks: implications for clinical trials and longitudinal tracking. Nat Aging. 2022;2:644–61. 10.1038/s43587-022-00248-2. 36277076 10.1038/s43587-022-00248-2 PMC9586209

[13] Belsky DW, Caspi A, Corcoran DL, et al. DunedinPACE, a DNA methylation biomarker of the pace of aging. Elife. 2022;11:e73420. 10.7554/eLife.73420. 35029144 10.7554/eLife.73420 PMC8853656

[14] Chen BH, Marioni RE, Colicino E, et al. DNA methylation-based measures of biological age: meta-analysis predicting time to death. Aging. 2016;8:1844–65. 10.18632/aging.101020. 27690265 10.18632/aging.101020 PMC5076441

[15] Marioni RE, Shah S, McRae AF, et al. DNA methylation age of blood predicts all-cause mortality in later life. Genome Biol. 2015;16:25. 10.1186/s13059-015-0584-6. 25633388 10.1186/s13059-015-0584-6 PMC4350614

[16] Stevenson AJ, McCartney DL, Harris SE, et al. Trajectories of inflammatory biomarkers over the eighth decade and their associations with immune cell profiles and epigenetic ageing. Clin Epigenetics. 2018;10:159. 10.1186/s13148-018-0585-x. 30572949 10.1186/s13148-018-0585-x PMC6302523

[17] Huang R-C, Lillycrop KA, Beilin LJ, et al. Epigenetic age acceleration in adolescence associates with BMI, inflammation, and risk score for middle age cardiovascular disease. J Clin Endocrinol Metab. 2019;104:3012–24. 10.1210/jc.2018-02076. 30785999 10.1210/jc.2018-02076 PMC6555851

[18] Irvin MR, Aslibekyan S, Do A, et al. Metabolic and inflammatory biomarkers are associated with epigenetic aging acceleration estimates in the GOLDN study. Clin Epigenetics. 2018;10:56. 10.1186/s13148-018-0481-4. 29713391 10.1186/s13148-018-0481-4 PMC5907301

[19] Feinleib M, Kannel WB, Garrison RJ, et al. The framingham offspring study. Design and preliminary data. Prev Med. 1975;4:518–25. 10.1016/0091-7435(75)90037-7. 1208363 10.1016/0091-7435(75)90037-7

[20] Fang Y, Doyle MF, Chen J, et al. Circulating immune cell phenotypes are associated with age, sex, CMV, and smoking status in the Framingham Heart Study offspring participants. Aging. 2023;15:3939–66. 10.18632/aging.204686. 37116193 10.18632/aging.204686 PMC10258017

[21] Ramasubramanian R, Meier HCS, Vivek S, et al. Evaluation of T-cell aging-related immune phenotypes in the context of biological aging and multimorbidity in the health and retirement study. Immun Ageing. 2022;19:33. 10.1186/s12979-022-00290-z. 35858901 10.1186/s12979-022-00290-z PMC9297609

[22] Thyagarajan B, Faul J, Vivek S, et al. Age-related differences in T-cell subsets in a nationally representative sample of people older than age 55: findings from the health and retirement study. The Journals of Gerontology: Series A. 2022;77:927–33. 10.1093/gerona/glab300. 10.1093/gerona/glab300 PMC9071411 34633448

[23] Griffiths P, Reeves M. Pathogenesis of human cytomegalovirus in the immunocompromised host. Nat Rev Microbiol. 2021;19:759–73. 10.1038/s41579-021-00582-z. 34168328 10.1038/s41579-021-00582-z PMC8223196

[24] Wertheimer AM, Bennett MS, Park B, et al. Aging and cytomegalovirus infection differentially and jointly affect distinct circulating T cell subsets in humans. J Immunol. 2014;192:2143–55. 10.4049/jimmunol.1301721. 24501199 10.4049/jimmunol.1301721 PMC3989163

[25] Chen J, Doyle MF, Fang Y, et al. Peripheral inflammatory biomarkers are associated with cognitive function and dementia: Framingham Heart Study offspring cohort. Aging Cell. 2023;22:e13955. 10.1111/acel.13955. 37584418 10.1111/acel.13955 PMC10577533

[26] Kuan P-F, Clouston S, Yang X, et al. Molecular linkage between post-traumatic stress disorder and cognitive impairment: a targeted proteomics study of World Trade Center responders. Transl Psychiatry. 2020;10:269. 10.1038/s41398-020-00958-4. 32753605 10.1038/s41398-020-00958-4 PMC7403297

[27] Wang M, Li Y, Lai M, et al. Alcohol consumption and epigenetic age acceleration across human adulthood. Aging. 2023. 10.18632/aging.205153. 37889500 10.18632/aging.205153 PMC10637803

[28] Bartlett DB, Firth CM, Phillips AC, et al. The age-related increase in low-grade systemic inflammation (Inflammaging) is not driven by cytomegalovirus infection. Aging Cell. 2012;11:912–5. 10.1111/j.1474-9726.2012.00849.x. 22708923 10.1111/j.1474-9726.2012.00849.x

[29] D’Agostino RB, Vasan RS, Pencina MJ, et al. General cardiovascular risk profile for use in primary care: the Framingham Heart Study. Circulation. 2008;117:743–53. 10.1161/CIRCULATIONAHA.107.699579. 18212285 10.1161/CIRCULATIONAHA.107.699579

[30] Klein SL, Flanagan KL. Sex differences in immune responses. Nat Rev Immunol. 2016;16:626–38. 10.1038/nri.2016.90. 27546235 10.1038/nri.2016.90

[31] Shen X, Wang C, Zhou X, et al. Nonlinear dynamics of multi-omics profiles during human aging. Nat Aging. 2024;4:1619–34. 10.1038/s43587-024-00692-2. 39143318 10.1038/s43587-024-00692-2 PMC11564093

[32] Benjamini Y, Hochberg Y. Controlling the false discovery rate: a practical and powerful approach to multiple testing. J R Stat Soc Ser B Stat Methodol. 1995;57:289–300. 10.1111/j.2517-6161.1995.tb02031.x.

[33] Therneau TM (2024) coxme: Mixed effects cox models. R package version 2.2-22, https://CRAN.R-project.org/package=coxme.

[34] Mittelbrunn M, Kroemer G. Hallmarks of T cell aging. Nat Immunol. 2021;22:687–98. 10.1038/s41590-021-00927-z. 33986548 10.1038/s41590-021-00927-z

[35] Nikolich-Žugich J. The twilight of immunity: emerging concepts in aging of the immune system. Nat Immunol. 2018;19:10–9. 10.1038/s41590-017-0006-x. 29242543 10.1038/s41590-017-0006-x

[36] Goronzy JJ, Weyand CM. Successful and maladaptive T cell aging. Immunity. 2017;46:364–78. 10.1016/j.immuni.2017.03.010. 28329703 10.1016/j.immuni.2017.03.010 PMC5433436

[37] The Milieu Intérieur Consortium, Patin E, Hasan M, et al. Natural variation in the parameters of innate immune cells is preferentially driven by genetic factors. Nat Immunol. 2018;19:302–14. 10.1038/s41590-018-0049-7. 29476184 10.1038/s41590-018-0049-7

[38] Oblak L, Van Der Zaag J, Higgins-Chen AT, et al. A systematic review of biological, social and environmental factors associated with epigenetic clock acceleration. Ageing Res Rev. 2021;69:101348. 10.1016/j.arr.2021.101348. 33930583 10.1016/j.arr.2021.101348

[39] Sánchez-Cabo F, Fuster V, Silla-Castro JC, et al. Subclinical atherosclerosis and accelerated epigenetic age mediated by inflammation: a multi-omics study. Eur Heart J. 2023;44:2698–709. 10.1093/eurheartj/ehad361. 37339167 10.1093/eurheartj/ehad361 PMC10393076

