# Supplementary material for: Immune cells and inflammatory proteins are differentially associated with subsequent DNA methylation biological aging measures in the Framingham Heart Study Offspring Cohort
Source: GeroScience. 2025 Oct 2;48(3):4373–94. doi: 10.1007/s11357-025-01883-4 (PMC13356187; doi:10.1007/s11357-025-01883-4)
Supplement: Supplementary file 2 — (PDF 716 KB) [file 11357_2025_1883_MOESM2_ESM.pdf]

Online Resource 2: Supplementary Figures
Rotti et al, Geroscience
Corresponding Author: Kathryn Lunetta, Boston University klunetta@bu.edu

Fig s1. Pearson correlation plot for the immune cell phenotypes

Fig s2. Pearson correlation plot for the inflammatory proteins

Fig s3. Pearson correlation plot for the DNAm aging metrics

Fig s4. Forest plots for the prospective associations between the immune cell phenotypes and all six DNAm aging outcomes in the full sample and all strata, Model 1 covariates, for the 27 immune cell phenotypes with significant associations with one of the outcomes in either the full sample or at least one of the strata

Fig s5. Forest plots for the prospective associations between the immune cell phenotypes and all six DNAm aging outcomes in the full sample and all strata, Model 2 covariates, for the 27 immune cell phenotypes with significant associations with one of the outcomes in either the full sample or at least one stratum.

Fig s6. Forest plots for the prospective associations between inflammatory protein biomarkers and all six DNAm aging outcomes in the full sample and all strata, Model 1 covariates, for the 56 inflammatory biomarkers with significant associations with one of the outcomes in the full sample or at least one stratum.

Fig s7. Forest plots for the prospective associations between inflammatory protein biomarkers and all six DNAm aging outcomes in the full sample and all strata, Model 2 covariates, for the 56 inflammatory biomarkers with significant associations with one of the outcomes in the full sample or at least one stratum.

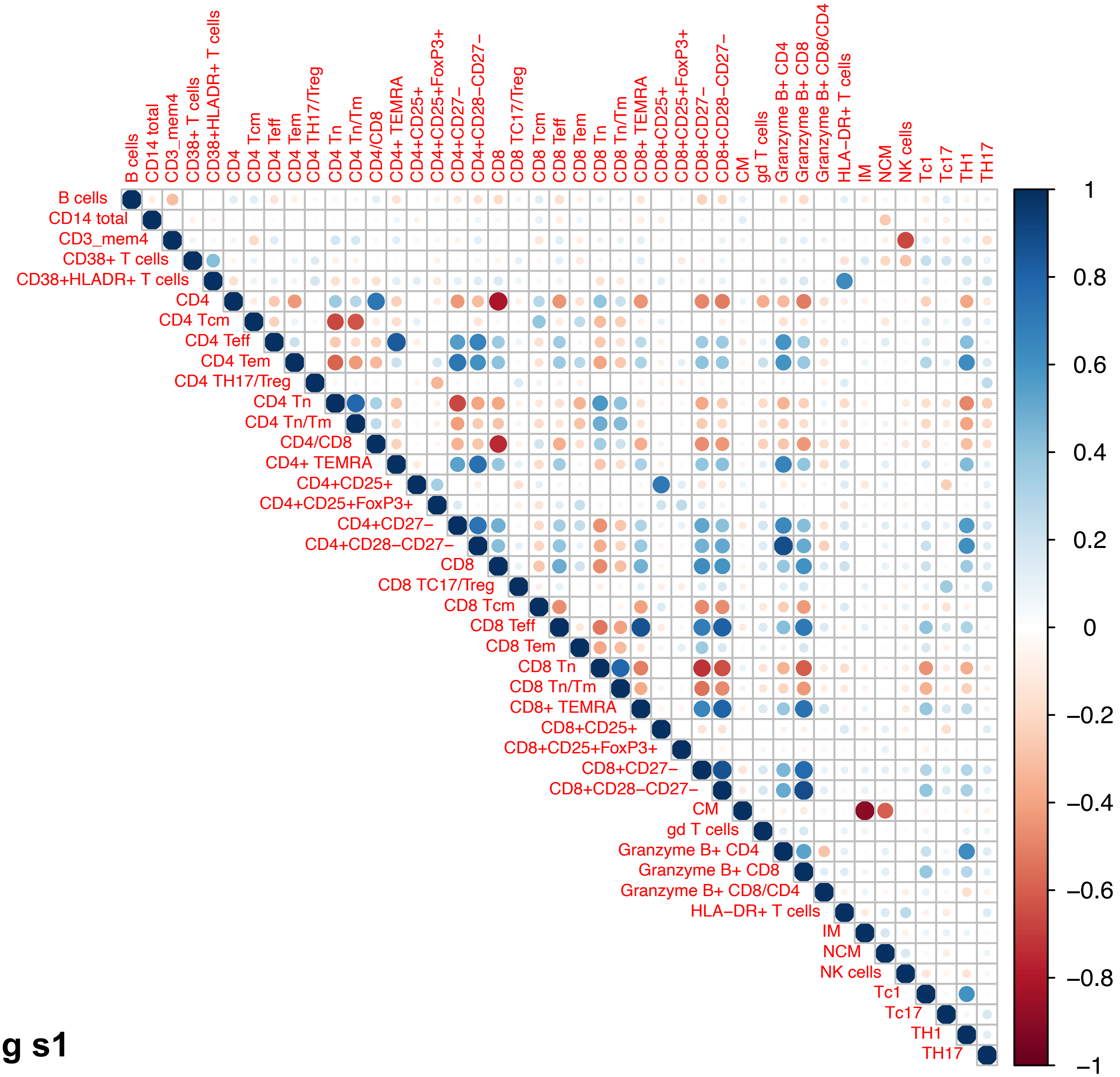
B cells
CD14 total
CD3_mem4
CD38+ T cells
CD38+HLADR+ T cells
CD4
CD4 Tcm
CD4 Teff
CD4 Tem
CD4 TH17/Treg
CD4 Tn
CD4 Tn/Tm
CD4/CD8
CD4+ TEMRA
CD4+CD25+
CD4+CD25+FoxP3+
CD4+CD27−
CD4+CD28−CD27−
CD8
CD8 TC17/Treg
CD8 Tcm
CD8 Teff
CD8 Tem
CD8 Tn
CD8 Tn/Tm
CD8+ TEMRA
CD8+CD25+
CD8+CD25+FoxP3+
CD8+CD27−
CD8+CD28−CD27−
CM
gd T cells
Granzyme B+ CD4
Granzyme B+ CD8
Granzyme B+ CD8/CD4
HLA−DR+ T cells
IM
NCM
NK cells
Tc1
Tc17
TH1
TH17
1
0.8
0.6
0.4
0.2
0
−0.2
−0.4
−0.6
−0.8
−1

Fig s1

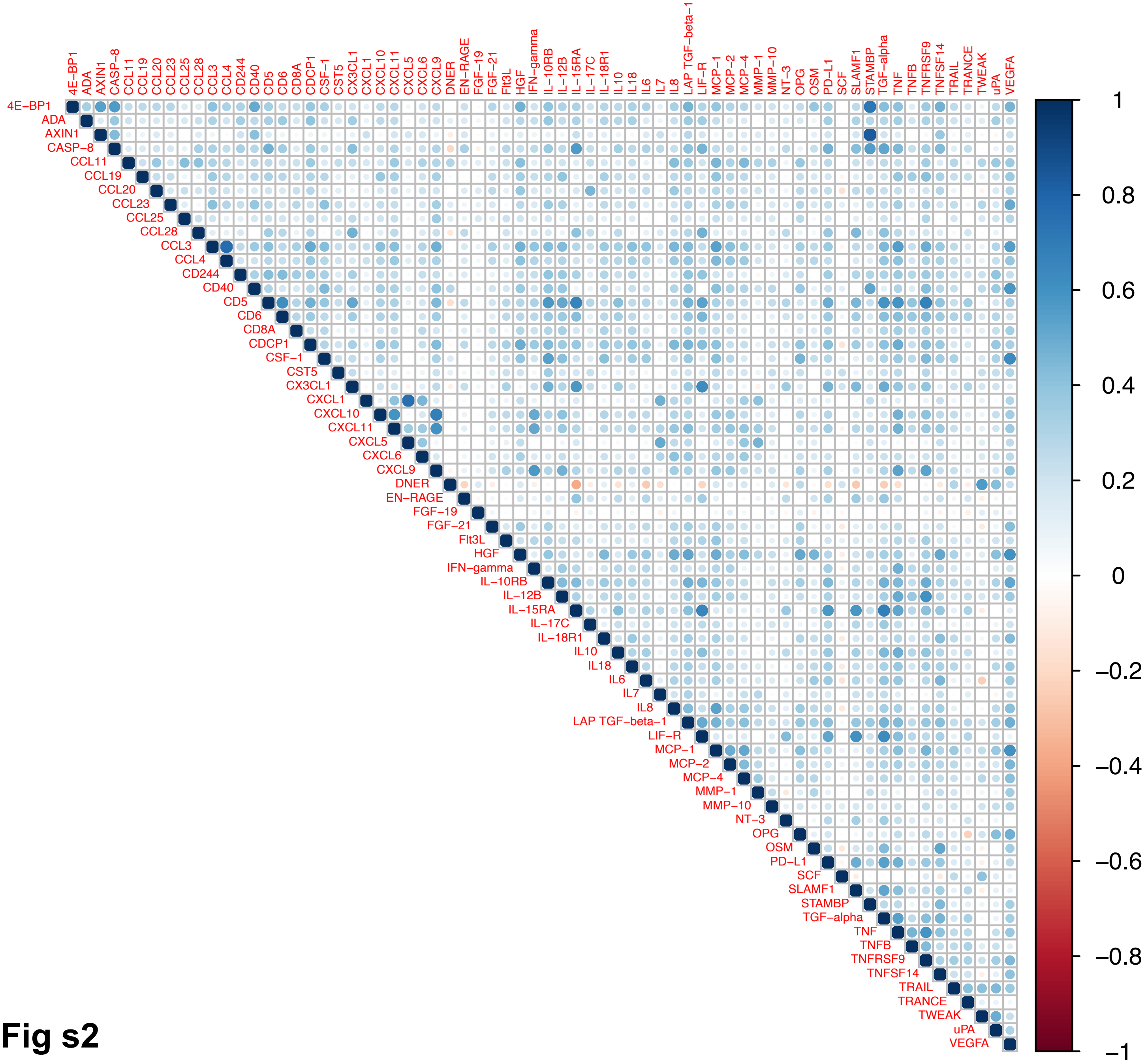
4E-BP1
ADA
AXIN1
CASP-8
CCL11
CCL19
CCL20
CCL23
CCL25
CCL28
CCL3
CCL4
CD244
CD40
CD5
CD6
CD8A
CDCP1
CSF-1
CST5
CX3CL1
CXCL1
CXCL10
CXCL11
CXCL5
CXCL6
CXCL9
DNER
EN-RAGE
FGF-19
FGF-21
Flt3L
HGF
IFN-gamma
IL-10RB
IL-12B
IL-15RA
IL-17C
IL-18R1
IL10
IL18
IL6
IL7
IL8
LAP TGF-beta-1
LIF-R
MCP-1
MCP-2
MCP-4
MMP-1
MMP-10
NT-3
OPG
OSM
PD-L1
SCF
SLAMF1
STAMBP
TGF-alpha
TNF
TNFB
TNFRSF9
TNFSF14
TRAIL
TRANCE
TWEAK
uPA
VEGFA
1
0.8
0.6
0.4
0.2
0
−0.2
−0.4
−0.6
−0.8
−1

Fig s2

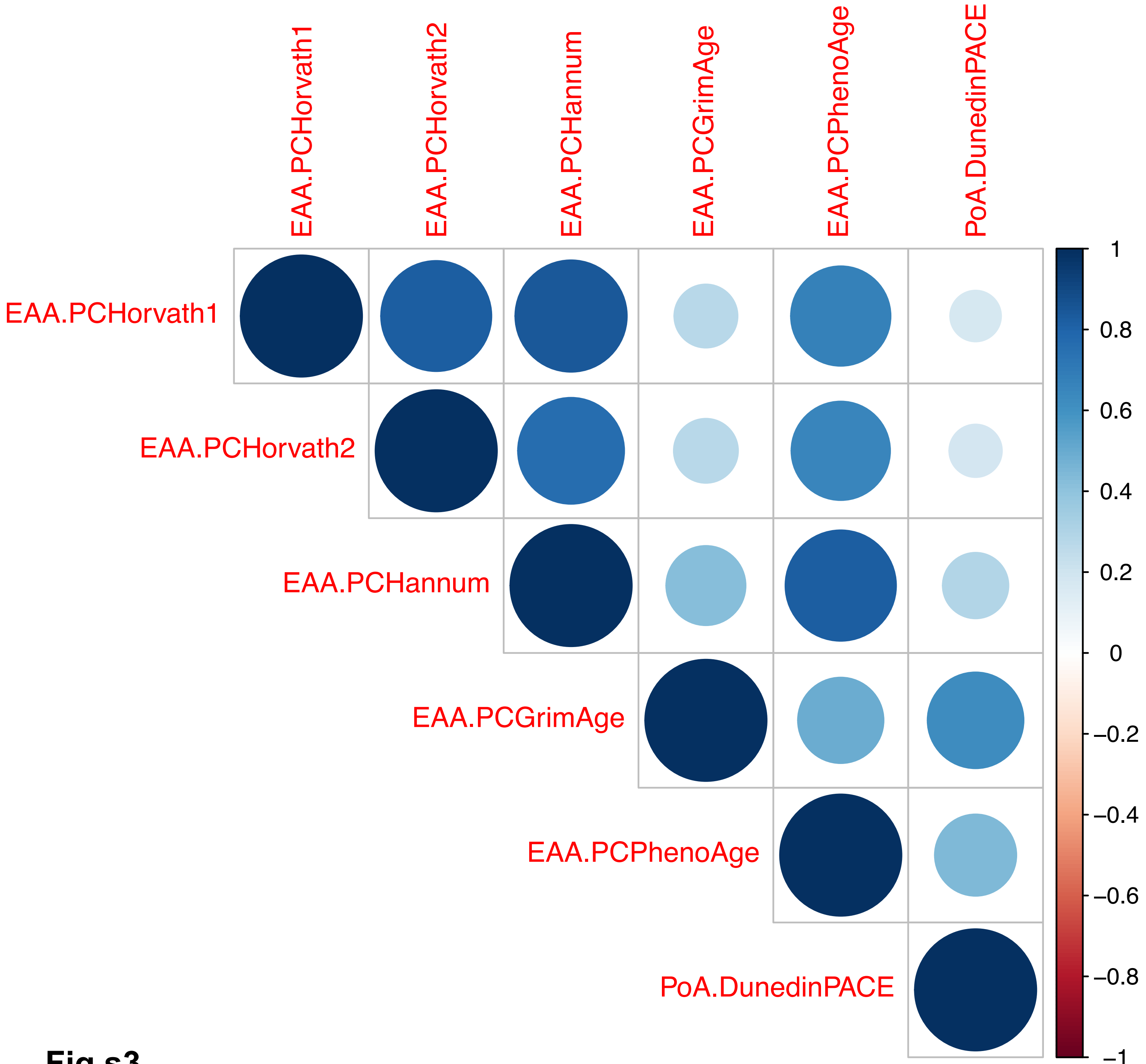
EAA.PCHorvath1
EAA.PCHorvath2
EAA.PCHannum
EAA.PCGrimAge
EAA.PCPhenoAge
PoA.DunedinPACE
EAA.PCHorvath1
EAA.PCHorvath2
EAA.PCHannum
EAA.PCGrimAge
EAA.PCPhenoAge
PoA.DunedinPACE
1
0.8
0.6
0.4
0.2
0
−0.2
−0.4
−0.6
−0.8
−1

Fig s3

Fig s4

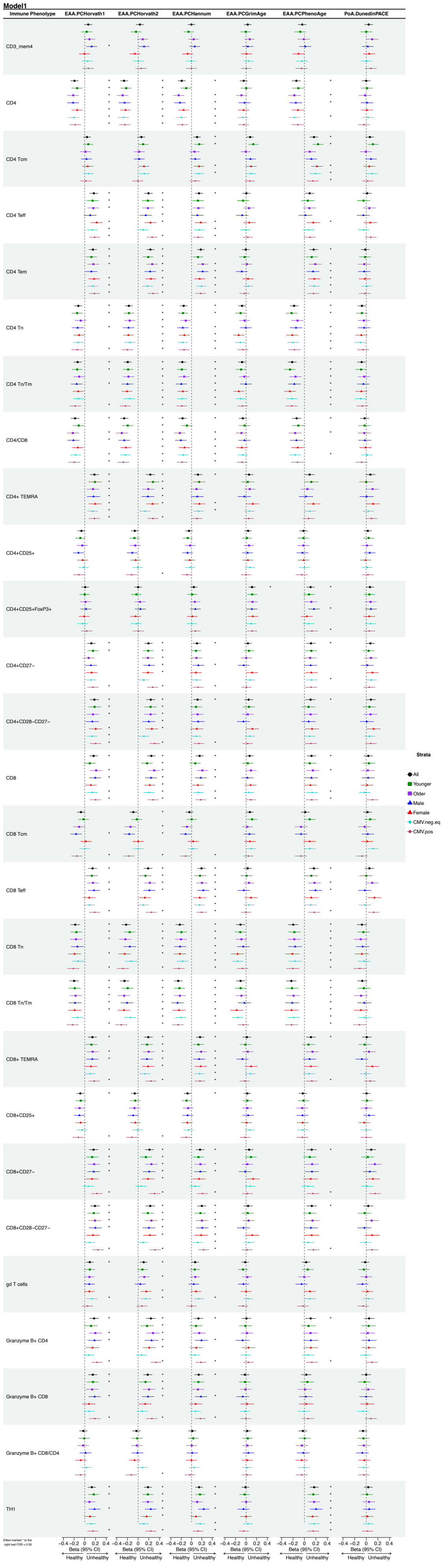
Model1
Immune Phenotype
EAA.PCHorvath1
EAA.PCHorvath2
EAA.PCHannum
EAA.PCGrimAge
EAA.PCPhenoAge
PoA.DunedinPACE
CD3_mem4
CD4
CD4 Tcm
CD4 Teff
CD4 Tem
CD4 Tn
CD4 Tn/Tm
CD4/CD8
CD4+ TEMRA
CD4+CD25+
CD4+CD25+FoxP3+
CD4+CD27−
CD4+CD28−CD27−
CD8
CD8 Tcm
CD8 Teff
CD8 Tn
CD8 Tn/Tm
CD8+ TEMRA
CD8+CD25+
CD8+CD27−
CD8+CD28−CD27−
gd T cells
Granzyme B+ CD4
Granzyme B+ CD8
Granzyme B+ CD8/CD4
TH1
Effect marked * to the right had FDR < 0.05
−0.4 −0.2 0 0.2 0.4
Beta (95% CI)
Healthy
Unhealthy
Strata
All
Younger
Older
Male
Female
CMV.neg.eq
CMV.pos

Fig s5

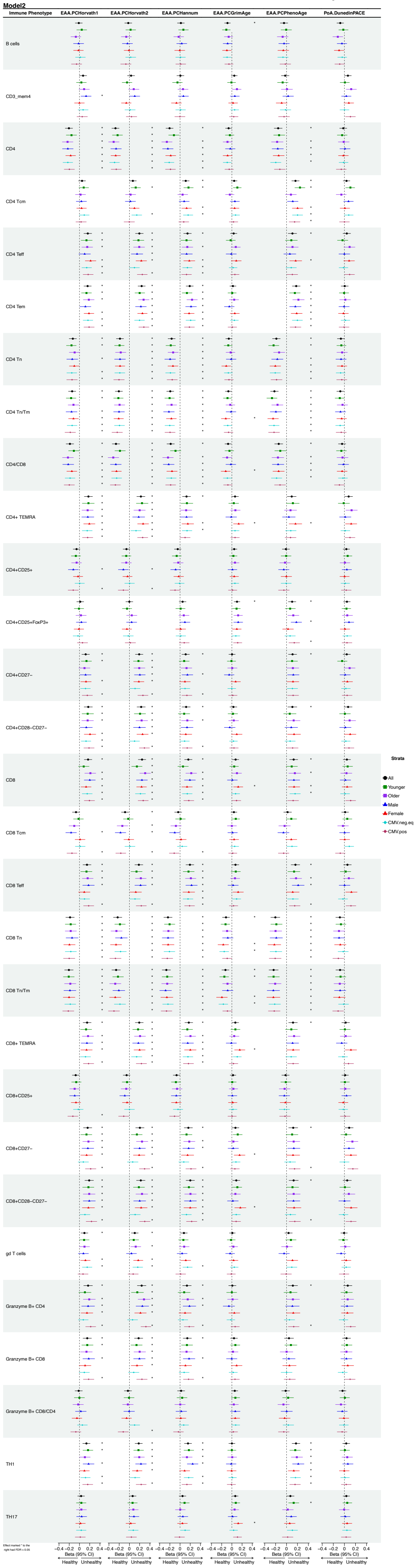
Model2
Immune Phenotype
EAA.PCHorvath1
EAA.PCHorvath2
EAA.PCHannum
EAA.PCGrimAge
EAA.PCPhenoAge
PoA.DunedinPACE
B cells
CD3_mem4
CD4
CD4 Tcm
CD4 Teff
CD4 Tem
CD4 Tn
CD4 Tn/Tm
CD4/CD8
CD4+ TEMRA
CD4+CD25+
CD4+CD25+FoxP3+
CD4+CD27−
CD4+CD28−CD27−
CD8
CD8 Tcm
CD8 Teff
CD8 Tn
CD8 Tn/Tm
CD8+ TEMRA
CD8+CD25+
CD8+CD27−
CD8+CD28−CD27−
gd T cells
Granzyme B+ CD4
Granzyme B+ CD8
Granzyme B+ CD8/CD4
TH1
TH17
Effect marked * to the right had FDR < 0.05
−0.4 −0.2 0 0.2 0.4
Beta (95% CI)
Healthy Unhealthy
Strata
All
Younger
Older
Male
Female
CMV.neg.eq
CMV.pos

Fig s6

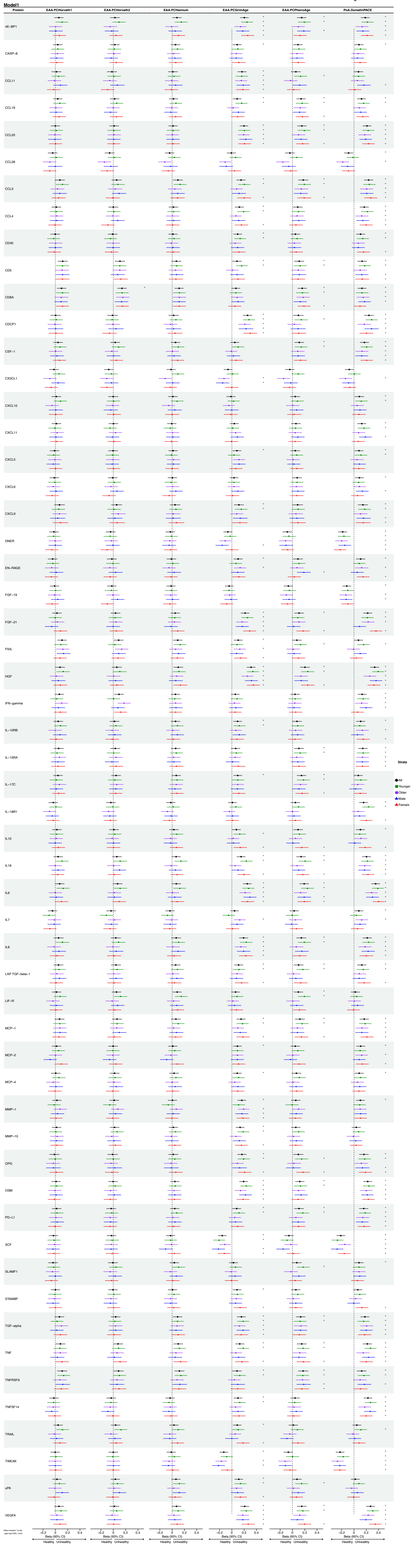

Model1
Protein
EAA.PCHorvath1
EAA.PCHorvath2
EAA.PCHannum
EAA.PCGrimAge
EAA.PCPhenoAge
PoA.DunedinPACE
4E−BP1
CASP−8
CCL11
CCL19
CCL20
CCL28
CCL3
CCL4
CD40
CD5
CD8A
CDCP1
CSF−1
CX3CL1
CXCL10
CXCL11
CXCL5
CXCL6
CXCL9
DNER
EN−RAGE
FGF−19
FGF−21
Flt3L
HGF
IFN−gamma
IL−10RB
IL−15RA
IL−17C
IL−18R1
IL10
IL18
IL6
IL7
IL8
LAP TGF−beta−1
LIF−R
MCP−1
MCP−2
MCP−4
MMP−1
MMP−10
OPG
OSM
PD−L1
SCF
SLAMF1
STAMBP
TGF−alpha
TNF
TNFRSF9
TNFSF14
TRAIL
TWEAK
uPA
VEGFA
Effect marked * to the right had FDR < 0.05
−0.2
0
0.2
0.4
Beta (95% CI)
Healthy
Unhealthy
Strata
All
Younger
Older
Male
Female

Fig s7

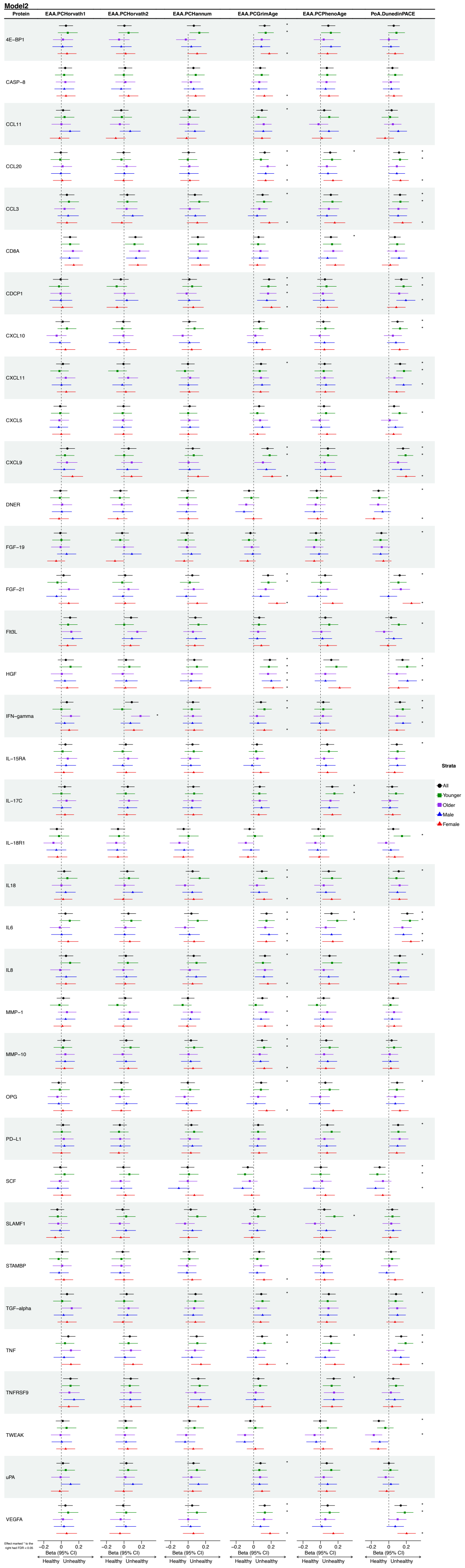

Model2
Protein
EAA.PCHorvath1
EAA.PCHorvath2
EAA.PCHannum
EAA.PCGrimAge
EAA.PCPhenoAge
PoA.DunedinPACE
4E−BP1
CASP−8
CCL11
CCL20
CCL3
CD8A
CDCP1
CXCL10
CXCL11
CXCL5
CXCL9
DNER
FGF−19
FGF−21
Flt3L
HGF
IFN−gamma
IL−15RA
IL−17C
IL−18R1
IL18
IL6
IL8
MMP−1
MMP−10
OPG
PD−L1
SCF
SLAMF1
STAMBP
TGF−alpha
TNF
TNFRSF9
TWEAK
uPA
VEGFA
Strata
All
Younger
Older
Male
Female
Effect marked * to the right had FDR < 0.05
−0.2
0
0.2
Beta (95% CI)
Healthy
Unhealthy
